# Risk of depression after new-onset dementia or cognitive impairment: the Health and Retirement Study

**DOI:** 10.1093/geroni/igag074

**Published:** 2026-06-30

**Authors:** Jingkai Wei, Youngran Kim, Victoria L Tang, Casey Crump

**Affiliations:** Department of Family and Community Medicine, McGovern Medical School, University of Texas Health Science Center at Houston, Houston, Texas, United States; Department of Epidemiology, School of Public Health, University of Texas Health Science Center at Houston, Houston, Texas, United States; Department of Management, Policy & Community Health, School of Public Health, University of Texas Health Science Center at Houston, Houston, Texas, United States; Joan and Stanford Alexander Division of Geriatric and Palliative Medicine, Department of Internal Medicine, McGovern Medical School, University of Texas Health Science Center at Houston, Houston, Texas, United States; Department of Family and Community Medicine, McGovern Medical School, University of Texas Health Science Center at Houston, Houston, Texas, United States; Department of Epidemiology, School of Public Health, University of Texas Health Science Center at Houston, Houston, Texas, United States

**Keywords:** Cognition, Mental health, Epidemiology

## Abstract

**Background and Objectives:**

The risk of depression following newly occurred dementia has not been well studied, particularly in diverse populations. This study aimed to estimate the relative risk of depression after newly occurred dementia or cognitive impairment in a cohort of adults aged ≥50 years.

**Research Design and Methods:**

Participants from the Health and Retirement Study with and without newly occurred dementia or cognitive impairment, and free of depression at baseline, were included. Dementia and cognitive impairment were ascertained using the Langa-Weir Classification of cognition, and depression was defined as ≥3 symptoms on the 8-item Center for Epidemiologic Studies Depression Scale. Pooled logistic regression with inverse probability weighting was used to estimate the cumulative incidence of depression following newly occurred dementia and cognitive impairment for up to 10 years. Risk ratios (RRs) with 95% confidence intervals (CIs) were obtained using 1,000 bootstrap samples. Subgroup analyses were conducted by age, sex, and race/ethnicity.

**Results:**

Risk of depression was significantly elevated after 2 years in those with newly occurred dementia (RR = 1.62, 95% CI: 1.17–2.03) or newly occurred cognitive impairment (RR = 1.62, 95% CI: 1.45–1.79). Elevated risks persisted throughout the 10-year follow-up. These risks were similar in men and women and significantly elevated in most subgroups.

**Discussion and Implications:**

Incident dementia and cognitive impairment were associated with immediate and sustained elevated risk of depression across 10 years of follow-up. These findings highlight the importance of prevention strategies and timely screening for depression in people with newly occurred dementia or cognitive impairment.

Innovation and Translational Significance: Among diverse U.S. older adults in the Health and Retirement Study, we use multiple time zeros and inverse probability-weighted models to estimate 10-year depression risk after new-onset dementia or cognitive impairment. We show depression risk increases within 2 years and remains elevated for a decade. Results support integrating routine depression screening and stepped care referral into cognitive assessment and dementia care, and inform health system and public health policies that fund prevention and community supports, improving quality of life in later life and reducing disability and caregiver burden.

Dementia affects more than 7 million U.S. adults aged 65 years and older, with numbers expected to rise in the coming decades (Alzheimer’s Association, 2024). For individuals diagnosed with dementia, reducing the risk of complications and improving quality of life are critical priorities (Wehrmann et al., 2021). Depression is one of the most common and burdensome comorbidities (Kitching, 2015), occurring in an estimated 20%–30% of people with Alzheimer’s disease and at even higher rates among those with vascular dementia (Enache et al., 2011). Beyond cognitive decline, dementia is frequently accompanied by neuropsychiatric symptoms, also referred to as behavioral and psychological symptoms of dementia (BPSD). Behavioral and psychological symptoms of dementia BPSD may include disturbances of mood, behavior, perception, and thought content, such as depression, apathy, anxiety, irritability, agitation, aggression, delusions, hallucinations, and sleep or appetite changes (Lyketsos et al., 2002). Depression/depressive symptoms are therefore often conceptualized as one of the common affective manifestations of BPSD (van der Linde et al., 2014), while also being studied in the broader literature as a risk factor (Diniz et al., 2013), prodromal manifestation (Berger et al., 1999; Brommelhoff et al., 2009), or sequela of dementia (Crump et al., 2024). Its co-occurrence with dementia may accelerate cognitive decline (Sekhon & Marwaha, 2020) and increase all-cause mortality (Petersen et al., 2017).

Most prior research has focused on the prevalence of depression in relatively homogeneous populations, without examining the timing of depression onset in relation to dementia diagnosis (Asmer et al., 2018). As a result, it was unable to distinguish preexistent from incident depression, thus limiting the relevance of findings for prevention and early intervention. A recent study based on a large Swedish registry found that individuals with newly diagnosed dementia had more than double the risk of developing depression compared with those without dementia (Crump et al., 2024). However, how findings may compare in more racially and ethnically diverse populations is unknown. Moreover, the risk of depression may also increase prior to dementia diagnosis, as milder forms of cognitive impairment have been associated with elevated depressive symptoms (Guo et al., 2023; Mirza et al., 2017). Clarifying the timing and magnitude of depression risk following newly occurred dementia or cognitive impairment in more diverse, representative populations is therefore crucial.

Evaluating heterogeneity across demographic groups is also important. Age may influence the association between cognitive decline and depression because earlier-onset cognitive impairment may have distinct psychosocial consequences, whereas depression detection in older ages may be complicated by comorbidity, somatic symptoms, and competing risks. Depression and dementia-related neuropsychiatric symptoms also may differ between men and women, with prior studies reporting sex differences in depressive symptoms among people with Alzheimer’s disease and dementia (Eikelboom et al., 2022). In addition, dementia burden, depressive symptom severity, diagnosis, treatment access, and social determinants of health differ across racial and ethnic groups in the United States (Vyas et al., 2020). Therefore, determining whether depression risk after newly occurred dementia or cognitive impairment varies according to age, sex, and race/ethnicity subgroups may help inform clinical screening and equity-focused prevention efforts.

To address these knowledge gaps, we sought to estimate the relative risk of depression after newly occurred dementia or cognitive impairment in a nationally representative longitudinal cohort of U.S. late-midlife and late-life adults with 10 years of follow-up, and to assess whether these associations were consistent across clinically relevant subgroups defined by age, sex, and race/ethnicity.

## Method

We used data from the Health and Retirement Study (HRS), a nationally representative longitudinal cohort of adults aged ≥50 years. Participants were first enrolled in 1992 and completed biennial questionnaires thereafter (Sonnega et al., 2014). There were new participants added to the HRS every 6 years, including the following birth cohorts: the original HRS, born in 1931–1941, starting from 1992; the Assets and Health Dynamics of the Oldest Old (AHEAD), born in 1924 or earlier, starting from 1993; the Children of the Depression (CODA), born in 1924–1930, starting from 1998; War Babies, born in 1942–1947, starting from 1998; Early baby boomers, born in 1948–1953, starting from 2004; and Mid baby boomers, born in 1954–1959, starting from 2010. We included participants aged ≥50 years at baseline who were followed up through Wave 14 (2018). Cognitive test-based dementia classification was available from Wave 3 (1996) onward, while depression data were available throughout follow-up. Therefore, cognitive status was assessed from Wave 3 through Wave 14. Depression data were available across HRS waves and were used to identify prior depression before analytic entry and incident depression during follow-up. Eligible participants were required to have no evidence of prevalent or prior depression, no dementia at baseline, and no important missing covariates. Among all participants aged ≥50 years in the original HRS cohort who attended Wave 4, we excluded individuals with dementia/cognitive impairment at the previous waves, depression at or prior to baseline, or missing covariate information (Supplementary Figures 1 and 2). All participants provided informed written consent, and the HRS datasets were fully de-identified.

### Multiple eligibility and time zeros

Time zero was defined as the wave when an individual either developed new-onset dementia/cognitive impairment or was free of dementia/cognitive impairment, with no dementia or depression prior to baseline. Individuals could have multiple time zeros. For example, an individual without dementia/cognitive impairment at Wave 4 and no depression at Wave 5 remained eligible for another time zero at Wave 5, until that individual experienced newly occurred dementia/cognitive impairment or depression, loss to follow-up, death, or the end of 10 years. Given the relatively small number of participants with newly occurred dementia/cognitive impairment, we used this approach to enable eligible individuals to have multiple time zeros (Supplementary Figures 3 and 4). This approach effectively increased statistical power without introducing bias (Caniglia et al., 2023).

### Assessment of dementia and cognitive impairment

Dementia and cognitive impairment were assessed at each wave using the established Langa-Weir Classification (Crimmins et al., 2011) based on three sets of cognitive tests: immediate and delayed 10-noun free recall test for memory (0–20 points), a serial sevens subtraction test for working memory (0–5 points), and a counting backward test for speed of mental processing (0–2 points). The total score therefore ranged from 0 to 27. Participants were categorized as normal (12–27 points), cognitively impaired but not demented (7–11 points), or demented (0–6 points). The Langa–Weir classification was previously validated with the Aging, Demographics, and Memory Study and had an accuracy rate of 87% for dementia categorization (Gianattasio et al., 2019). Cognitive impairment was defined as being either demented (0–6 points) or cognitively impaired but not demented (7–11 points). An individual was considered to have newly occurred dementia if his/her score dropped from >6 in a previous wave to ≤6 for the first time in the current wave, or having cognitive impairment if his/her score dropped from >11 in a previous wave to ≤11 for the first time in the current wave.

### Assessment of depression

Depression was assessed using the 8-item Center for Epidemiologic Studies Depression Scale (CES-D) at each wave. The eight items include “felt depressed,” “everything an effort,” “sleep was restless,” “was happy,” “felt lonely,” “felt sad,” “could not get going,” and “enjoyed life.” Participants indicated “Yes/No” for eight symptoms experienced in the past week. A “Yes” answer scored 1 point except for items “was happy” and “enjoyed life”, for which “No” scored 1 point. The total scores ranged from 0 to 8, and any individuals with a CES-D score ≥3 were categorized as having depression. This cutoff point has been validated against the World Health Organization’s Composite International Diagnostic Interview (CIDI-SF) with high sensitivity and specificity (Glymour et al., 2012). The 8-item CES-D has demonstrated good internal consistency in HRS/AHEAD, with Cronbach’s α ranging from 0.81 to 0.83 in HRS Waves 2–3 and from 0.77 to 0.79 in AHEAD Waves 1–2 (Steffick, 2000). The original 20-item CES-D has also shown high internal consistency, with Cronbach’s α approximately 0.85 in general-population samples and approximately 0.90 in a psychiatric patient sample (Radloff, 1977). Shortened CES-D measures have been used in prior population-based studies involving older adults with cognitive impairment or dementia, including the Washington Heights-Inwood Columbia Aging Project, which used the 10-item CES-D in analyses of late-life depression, mild cognitive impairment (MCI), and dementia (Richard et al., 2013). The eight-item CES-D was used by the English Longitudinal Study of Ageing to assess depression among participants with no impairment, mild cognitive impairment, and dementia (Beach et al., 2023) and by the Health and Retirement Study to examine bidirectional associations between depressive symptoms and MCI (Guo et al., 2023).

### Other covariates

Covariates were selected a priori based on prior literature and clinical plausibility as factors associated with both cognitive impairment/dementia and depression. These included demographic and social factors (including age, sex, race/ethnicity, education, and marital/partnered status), behavioral and anthropometric factors (including smoking and body mass index), and cardiometabolic/cerebrovascular comorbidities (including hypertension, diabetes, heart disease, and stroke). These factors have been associated with dementia or cognitive decline in prior evidence syntheses, including the Lancet Commission on dementia prevention (Livingston et al., 2024), and in research on depression risk among older adults and vascular risk factors (Kuźma et al., 2018). Race/ethnicity was included as a social variable reflecting differential distributions of social determinants, health care access, and vascular risk rather than as a biological construct. Death and the year of death were obtained from household members and were matched with the National Death Index.

### Statistical analysis

All subcohorts were merged for analysis. In descriptive analyses, characteristics were compared between participants with and without dementia or cognitive impairment using Chi-square and *t*-tests. We used an inverse probability-weighted model to estimate the risk of depression following newly occurred dementia or cognitive impairment (Robins et al., 2000). This approach was chosen because dementia and cognitive impairment were not randomly assigned, and participants who developed these conditions differed from those who did not with respect to demographic, socioeconomic, behavioral, and clinical factors that may also influence depression risk. Inverse probability weighting controls for confounding by using the estimated probability of each participant’s observed exposure and censoring history to create a weighted pseudo-population in which measured covariates are balanced between comparison groups. The marginal structural model then estimates the cumulative incidence of depression that would be expected in the observed exposure groups, while reducing bias from measured confounding and differential loss to follow-up. This approach is well suited for longitudinal observational data and has been widely used to estimate causal effects when randomization is not possible.

At each person-wave baseline, we estimated stabilized inverse probability weights for newly occurred dementia/cognitive impairment and for censoring using measured covariates. We then fit a weighted pooled logistic regression model for the interval-specific probability of depression among participants who were depression free at the prior wave. The model included dementia/cognitive impairment status, follow-up time, follow-up time squared, and interactions between dementia/cognitive impairment and follow-up time terms. Death was modeled as a competing risk. Within each group, survival was calculated by multiplying the predicted probability of remaining depression-free across follow-up intervals, and cumulative risk was defined as one minus this survival. Because HRS interviews were conducted biennially, 2 years was the earliest follow-up time at which incident depressive symptoms could be assessed after each person-wave baseline. We therefore used the 2-year estimate to characterize the earliest observable risk and the 10-year estimate to characterize the longest prespecified follow-up period available for evaluating sustained risk. Estimates at 4, 6, and 8 years were also reported to show the trajectory of cumulative depression risk over time. A total of 1,000 sets of bootstrapping were conducted to estimate the 95% confidence intervals (CIs). The associations were statistically significant if the 95% CIs for RRs did not include 1. The analysis was also conducted in different subgroups by age (<65 years, 65–74 years, ≥75 years), sex, and race/ethnicity (non-Hispanic White, other). All analyses were conducted using SAS 9.4 (Cary, NC).

## Results

### Baseline characteristics

For the comparison between participants with and without dementia, a total of 72,038 non-unique participants across all waves (15,893 unique participants) were included, comprising 1,602 with newly occurred dementia and 70,436 without dementia. Compared with those without dementia, participants with newly occurred dementia were older (mean age 80 vs 68 years), more likely to be non-Hispanic Black (19% vs 11%), and had lower levels of education (less than high school: 39% vs 14%; high school graduate or above: 58% vs 83%). They were also less likely to be married/partnered (47% vs 72%) or currently smoking (8% vs 11%), had a higher prevalence of hypertension (63% vs 53%), diabetes (24% vs 16%), heart disease (38% vs 22%), and stroke (26% vs 6%), and a lower mean BMI (25.3 vs 27.5) (Table 1). After applying inverse probability weighting for newly occurred dementia, all covariates were balanced between groups (absolute values of standardized mean difference below 1) (Supplementary Figure 5A).

**Table 1:** Baseline characteristics of participants

Variable	With dementia(*n* = 1,602)	Without dementia(*n* = 70,436)	With cognitive impairment(*n* = 4,073)	Without cognitive impairment(*n* = 54,267)
**Age, years**	**79.8 ± 10.6**	**67.6 ± 9.7**	**72.2 ± 10.9**	**66.2 ± 9.0**
**Women, *n* (%)**	841 (52.5)	35,290 (50.1)	**1,909 (46.9)**	**28,141 (51.9)**
**Race/ethnicity, *n* (%)**				
Non-Hispanic White	**1,151 (71.9)**	**56,991 (80.9)**	**2,984 (73.3)**	**45,815 (84.4)**
Non-Hispanic Black	**296 (18.5)**	**7,538 (10.7)**	**633 (15.5)**	**4,572 (8.4)**
Hispanic	**29 (1.8)**	**1,526 (2.2)**	**100 (2.5)**	**1,112 (2.1)**
Other	**126 (7.9)**	**4,381 (6.2)**	**356 (8.7)**	**2,768 (5.1)**
**Education, *n* (%)**				
Below high school	**628 (39.2)**	**9,552 (13.6)**	**1,052 (25.8)**	**4,849 (8.9)**
GED	**52 (3.3)**	**2,687 (3.8)**	**198 (4.9)**	**1,818 (3.4)**
High school graduate	**460 (28.7)**	**21,590 (30.7)**	**1,343 (33.0)**	**16,157 (29.8)**
Some college	**253 (15.8)**	**16,873 (24.0)**	**846 (20.8)**	**13,789 (25.4)**
College graduate or above	**209 (13.1)**	**19,734 (28.0)**	**634 (15.6)**	**17,654 (32.5)**
**Married or partnered, *n* (%)**	**752 (46.9)**	**50,824 (72.2)**	**2,608 (64.0)**	**40,241 (74.2)**
**Current smoking, *n* (%)**	**126 (7.9)**	**7,567 (10.7)**	**491 (12.1)**	**5,682 (10.5)**
**Body mass index, kg/m^2^**	**25.3 ± 5.3**	**27.5 ± 5.2**	**26.7 ± 5.2**	**27.5 ± 5.2**
**Hypertension, *n* (%)**	**1,010 (63.1)**	**37,057 (52.6)**	**2,403 (59.0)**	**27,157 (50.0)**
**Diabetes, *n* (%)**	**390 (24.3)**	**11,558 (16.4)**	**801 (19.7)**	**8,028 (14.8)**
**Heart disease, *n* (%)**	**600 (37.5)**	**15,111 (21.5)**	**1,156 (28.4)**	**10,642 (19.6)**
**Stroke, *n* (%)**	**423 (26.4)**	**3,998 (5.7)**	**463 (11.4)**	**2,487 (4.6)**

*Note.* GED = General Educational Development. Continuous variables are expressed as mean ± standard deviation. Values in bold indicate a statistically significant difference between the two groups (*p* < .05).

For the comparison between participants with and without cognitive impairment, a total of 58,340 non-unique participants (14,047 unique participants) were included, comprising 4,073 with newly occurred cognitive impairment and 54,267 without. Compared with those without cognitive impairment, participants with newly occurred cognitive impairment were older (72 vs 66 years), more predominantly male (53% vs 48%), and more likely to be non-Hispanic Black (16% vs 8%). They also had lower educational attainment (less than high school: 26% vs 9%; high school graduate or above: 69% vs 88%), were slightly less likely to be married or partnered (64% vs 74%), and had higher prevalence of smoking (12% vs 10%), hypertension (59% vs 50%), diabetes (20% vs 15%), heart disease (28% vs 20%), stroke (11% vs 5%), and a lower mean BMI (26.7 vs 27.5) (Table 1). After applying inverse probability weighting for newly occurred cognitive impairment, all covariates were balanced between groups (absolute values of standardized mean difference below 1) (Supplementary Figure 5B).

### Risk of depression following dementia

**Figure 1:**
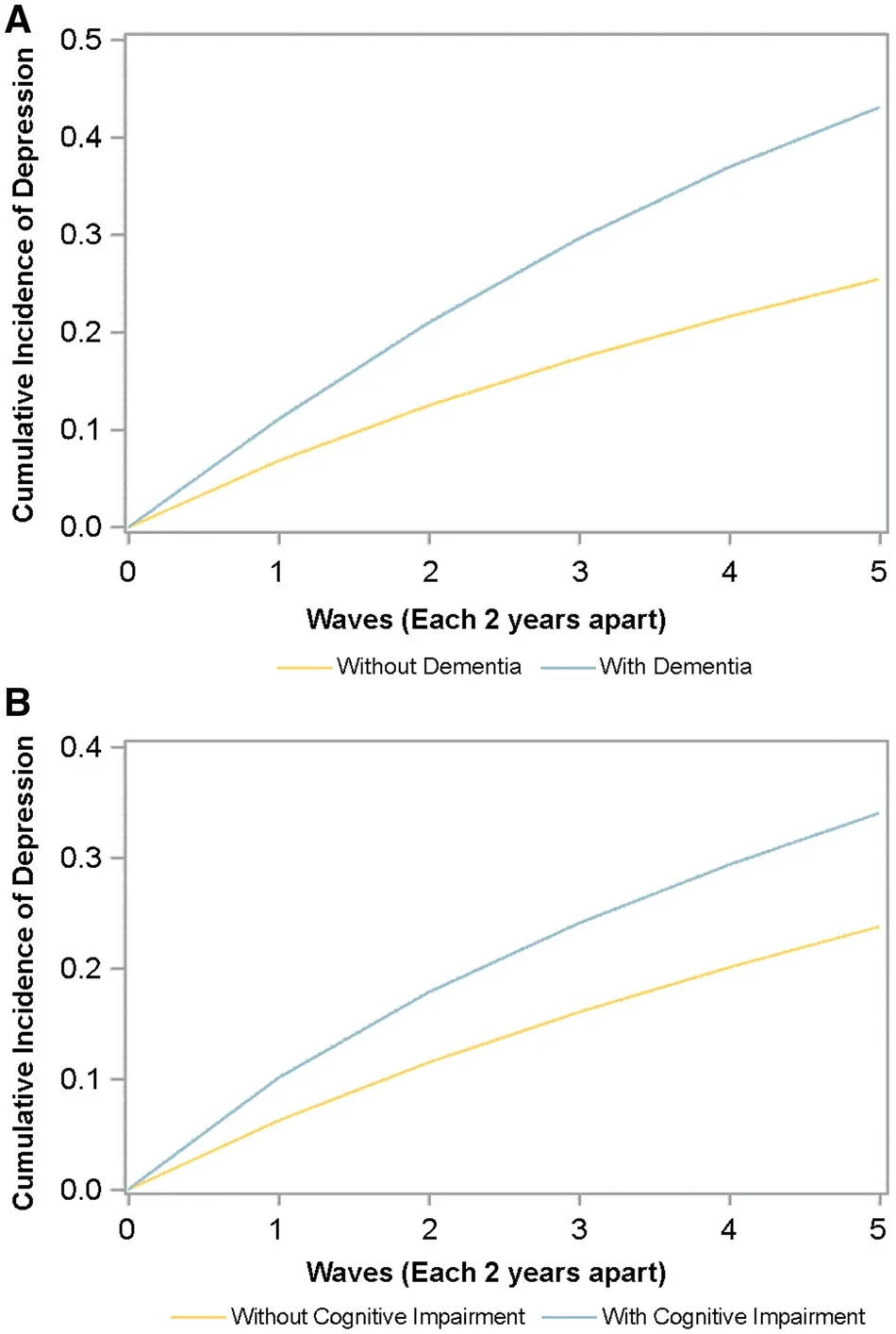
Cumulative incidence of depression during 10 years of follow-up among older adults with and without (A) newly occurred dementia and (B) newly occurred cognitive impairment.

The mean follow-up duration for the dementia comparison was 7.8 ± 5.1 years. During follow-up, 4,252 depression cases were reported (82 in participants with newly occurred dementia, 4,170 in those without). In addition, 95 with newly occurred dementia and 3,315 without were lost to follow-up, and 435 with dementia and 2,677 without dementia died without depression. Participants with newly occurred dementia had a higher cumulative risk of depression at the first available follow-up assessment 2 years later (risk ratio [RR] = 1.62, 95% CI: 1.17–2.03), and this elevated risk persisted through the maximum prespecified follow-up of 10 years (RR = 1.69, 95% CI: 1.39–2.00) (Figure 1A and Table 2).

**Table 2:** Subsequent risk of depression following newly occurred dementia or cognitive impairment

Length of follow-up	Cumulative incidence of depression			Cumulative incidence of cognitive impairment		
	People without dementia	People with newly occurred dementia	Risk ratio (95% CI)	People without cognitive impairment	People with newly occurred cognitive impairment	Risk ratio (95% CI)
**2 years**	0.07	0.11	**1.62 (1.17–2.03)**	0.06	0.10	**1.62 (1.45–1.79)**
**4 years**	0.13	0.21	**1.68 (1.32–2.00)**	0.11	0.18	**1.55 (1.43–1.67)**
**6 years**	0.17	0.30	**1.71 (1.39–2.02)**	0.16	0.24	**1.50 (1.40–1.60)**
**8 years**	0.22	0.37	**1.71 (1.40–2.04)**	0.20	0.29	**1.46 (1.37–1.55)**
**10 years**	0.25	0.43	**1.69 (1.39–2.00)**	0.24	0.34	**1.43 (1.35–1.52)**

*Note.* CI = confidence interval. Values in bold indicate statistically significant associations.

Stratified by age, among participants younger than 65 years, those with newly occurred dementia had a significantly higher risk of depression after 4 years (RR = 2.25, 95% CI: 1.25–3.07), which remained significant after 10 years (RR = 2.05, 95% CI: 1.47–2.58). Among those aged ≥75 years, those with newly occurred dementia had a higher risk of depression after 2 years (RR = 1.53, 95% CI: 1.07–1.89), which remained significant after 10 years (RR = 1.64, 95% CI: 1.28–1.94). No association was found among people aged between 65 and 74 years (Supplementary Figures 6 and Table 1, Table 3).

**Table 3:** Subsequent risk of depression following newly occurred dementia or cognitive impairment in different subgroups

Length of follow-up	By age			By sex		By race/ethnicity	
	Age < 65	**Age 65**–**74**	Age ≥75	Men	Women	Non-Hispanic White(*n* = 58,142)	Other races/ethnicities(*n* = 13,896)
	(*n* = 30,542)	(*n* = 22,632)	(*n* = 18,864)	(*n* = 35,907)	(*n* = 36,131)		
**Depression following newly occurred dementia**							
2 years	2.28 (0.99–3.41)	1.26 (0.45–1.96)	**1.53 (1.07–1.89)**	**1.79 (1.08–2.45)**	1.56 (0.91–2.17)	1.61 (0.95–2.24)	1.36 (0.76–1.86)
4 years	**2.25 (1.25–3.07)**	1.15 (0.52–1.74)	**1.71 (1.38–2.07)**	**1.82 (1.32–2.39)**	**1.66 (1.15–2.10)**	**1.73 (1.19–2.22)**	1.36 (0.94–1.75)
6 years	**2.19 (1.41–2.86)**	1.08 (0.55–1.61)	**1.77 (1.44–2.13)**	**1.81 (1.32–2.38)**	**1.73 (1.26–2.13)**	**1.80 (1.34–2.29)**	**1.36 (1.02–1.75)**
8 years	**2.13 (1.49–2.71)**	1.02 (0.57–1.49)	**1.74 (1.39–2.05)**	**1.77 (1.30–2.31)**	**1.76 (1.34–2.14)**	**1.81 (1.37–2.32)**	**1.38 (1.04–1.76)**
10 years	**2.05 (1.47–2.58)**	0.99 (0.59–1.39)	**1.64 (1.28–1.94)**	**1.70 (1.25–2.18)**	**1.77 (1.34–2.13)**	**1.79 (1.33–2.25)**	**1.41 (1.05–1.74)**
**Length of follow-up**	**Age < 65**	**Age 65**–**74**	**Age ≥75**	**Men**	**Women**	**Non-Hispanic White** **(*n* = 48,799)**	**Other races/ethnicities** **(*n* = 9,541)**
	**(*n* = 27,213)**	**(*n* = 18,684)**	**(*n* = 12,443)**	**(*n* = 28,290)**	**(*n* = 30,050)**		
**Depression following newly occurred cognitive impairment**							
2 years	**1.78 (1.48–2.06)**	**1.46 (1.17–1.74)**	**1.34 (1.15–1.53)**	**1.89 (1.64–2.16)**	**1.50 (1.28–1.74)**	**1.63 (1.42–1.84)**	**1.52 (1.27–1.76)**
4 years	**1.66 (1.44–1.86)**	**1.44 (1.21–1.64)**	**1.31 (1.18–1.44)**	**1.74 (1.55–1.93)**	**1.50 (1.34–1.67)**	**1.54 (1.39–1.71)**	**1.51 (1.31–1.70)**
6 years	**1.58 (1.40–1.75)**	**1.41 (1.24–1.58)**	**1.30 (1.19–1.43)**	**1.63 (1.48–1.79)**	**1.49 (1.36–1.63)**	**1.48 (1.35–1.62)**	**1.50 (1.33–1.67)**
8 years	**1.52 (1.36–1.67)**	**1.40 (1.25–1.55)**	**1.31 (1.20–1.43)**	**1.56 (1.43–1.70)**	**1.48 (1.35–1.60)**	**1.43 (1.32–1.56)**	**1.48 (1.32–1.65)**
10 years	**1.48 (1.33–1.62)**	**1.39 (1.26–1.52)**	**1.34 (1.23–1.46)**	**1.51 (1.39–1.64)**	**1.45 (1.33–1.57)**	**1.40 (1.29–1.52)**	**1.46 (1.31–1.62)**

*Note.* Values report risk ratios, with 95% confidence intervals in parentheses. Values in bold indicate statistically significant associations.

Stratified by sex, men with newly occurred dementia had a higher risk of depression after 2 years (RR = 1.79, 95% CI: 1.08–2.45), which remained significant after 10 years (RR = 1.70, 95% CI: 1.25–2.18). Women with newly occurred dementia had a significantly higher risk beginning at 4 years (RR = 1.66, 95% CI: 1.15–2.10), which persisted through 10 years (RR = 1.77, 95% CI: 1.34–2.13) (Supplementary Figure 7 and Table 2 and Table 3).

Stratified by race/ethnicity, non-Hispanic White participants with newly occurred dementia had a significantly higher risk of depression beginning at 4 years (RR = 1.73, 95% CI: 1.19–2.22), which remained significant after 10 years (RR = 1.79, 95% CI: 1.33–2.25). Participants of other races and ethnicities with newly occurred dementia had a significantly higher risk beginning at 6 years (RR = 1.36, 95% CI: 1.02–1.75), which persisted through 10 years (RR = 1.41, 95% CI: 1.05–1.74) (Supplementary Figure 8 and Table 2, Table 3).

### Risk of depression following cognitive impairment

The mean follow-up duration for the cognitive impairment comparison was 8.2 ± 5.2 years. During the period of follow-up, 3,315 depression cases were reported (308 in participants with newly occurred cognitive impairment, 3,007 in those without). In addition, 221 with cognitive impairment and 2,444 without were lost to follow-up, and 450 with cognitive impairment and 1,392 without died without depression. Participants with newly occurred cognitive impairment had a higher cumulative risk of depression at the first available follow-up assessment 2 years later (RR = 1.62, 95% CI: 1.45–1.79), and this elevated risk persisted through the maximum prespecified follow-up of 10 years (RR = 1.43, 95% CI: 1.35–1.52) (Figure 1B and Table 2).

Stratified by age, among participants younger than 65 years, those with newly occurred cognitive impairment had a higher risk of depression after 2 years (RR = 1.78, 95% CI: 1.48–2.06), which remained significant after 10 years (RR = 1.48, 95% CI: 1.33–1.62). Among those aged 65–74 years, those with newly occurred cognitive impairment had a higher risk of depression after 2 years (RR = 1.46, 95% CI: 1.17–1.74), which remained significant after 10 years (RR = 1.39, 95% CI: 1.26–1.52). Among those aged ≥75 years, those with newly occurred cognitive impairment had a higher risk of depression after 2 years (RR = 1.34, 95% CI: 1.15–1.53), which remained significant after 10 years (RR = 1.34, 95% CI: 1.23–1.46) (Supplementary Figure 9 and Table 4, Table 3).

Stratified by sex, men with newly occurred cognitive impairment had an elevated risk of depression after 2 years (RR = 1.89, 95% CI: 1.64–2.16), which persisted through 10 years (RR = 1.51, 95% CI: 1.39–1.64). Similarly, women with newly occurred cognitive impairment had a higher risk after 2 years (RR = 1.50, 95% CI: 1.28–1.74), which remained significant at 10 years (RR = 1.45, 95% CI: 1.33–1.57) (Supplementary Figure 10 and Table 5, Table 3).

Stratified by race/ethnicity, non-Hispanic White participants with newly occurred cognitive impairment had a higher risk of depression beginning at 2 years (RR = 1.63, 95% CI: 1.42–1.84), which remained significant after 10 years (RR = 1.40, 95% CI: 1.29–1.52). Participants of other races and ethnicities with newly occurred cognitive impairment had a significantly higher risk beginning at 2 years (RR = 1.52, 95% CI: 1.27–1.76), which persisted through 10 years (RR = 1.46, 95% CI: 1.31–1.62) (Supplementary Figure 11 and Table 6, Table 3).

## Discussion

In this nationally representative cohort of late-midlife and late-life adults, newly occurred dementia and cognitive impairment were associated with a sustained increased risk of depression during 10 years of follow-up, independent of covariates related to both conditions. These associations were similar in men and women and were observed in most other subgroups.

These findings should be interpreted within the broader framework of BPSD in dementia and cognitive impairment (Finkel et al., 1996; Lyketsos et al., 2002; van der Linde et al., 2014). Depression after newly occurred dementia or cognitive impairment should not be understood solely as a separate comorbid psychiatric disorder; it may also represent an affective neuropsychiatric manifestation of cognitive decline or underlying neurodegenerative and vascular pathology (Enache et al., 2011; Taylor et al., 2013). At the same time, depressive symptoms in this setting are clinically heterogeneous and may reflect overlapping mechanisms, including BPSD, major depressive disorder, prodromal symptoms, shared vascular or neurodegenerative pathways, psychosocial responses to functional decline, social isolation, or caregiver-related stressors (Olin et al., 2002). Thus, our findings support proactive recognition and management of new depressive symptoms after cognitive decline, regardless of whether they are ultimately classified clinically as BPSD, comorbid depression, or both.

Prospective research on dementia, cognitive impairment, and subsequent depression remains limited. In a national Swedish cohort, Crump et al. reported that patients with Alzheimer’s disease had an elevated risk of depression during a median follow-up of 8 years (adjusted hazard ratio = 2.38, 95% CI: 2.29–2.47), and even higher risk in the period shortly after dementia diagnosis (Crump et al., 2024), consistent with our findings. Similarly, in the Rotterdam Study, Mirza et al. observed that individuals with mild cognitive impairment had a higher risk of developing depressive disorder (odds ratio = 3.13, 95% CI: 1.26–7.77) (Mirza et al., 2017). Notably, both cohorts were European based, whereas our study drew on a more diverse, population-based U.S. sample. In addition, Guo et al., using HRS data, found that baseline mild cognitive impairment was associated with increased risk of depressive symptoms (hazard ratio = 1.16, 95% CI: 1.01–1.33) over 20 years (Guo et al., 2023), although that analysis was limited to prevalent cognitive impairment and did not account for the timing of depression onset. Our results extend this evidence by demonstrating that newly occurred dementia and cognitive impairment are associated with a higher risk of new-onset depression and thus providing a clearer timeframe for preventive or therapeutic intervention.

Several mechanisms may explain the elevated risk of depression. Cerebral small vessel disease and white-matter hyperintensities may disrupt frontostriatal-subcortical pathways, supporting the “vascular depression hypothesis,” a major model for late-life depression (Taylor et al., 2013). Also, pathology of Alzheimer’s disease, including tau and β-amyloid, may impair serotonergic and noradrenergic tone in hippocampal-limbic circuits, disrupting mood regulation (Chakraborty et al., 2019; Falgàs et al., 2024). In addition, reduced social engagement among people with dementia and cognitive impairment may further contribute to depression risk (Hackett et al., 2019; Luo, 2023).

We also found that the association between either dementia or cognitive impairment and subsequent depression was significant among both men and women. For dementia, the associations among men started immediately after diagnosis and with a larger magnitude than among women. This is a similar pattern as the Swedish study, which reported significant associations for both sexes, with stronger associations in men (Crump et al., 2024). Although the overall prevalence of depression is higher among women in the general population, previous findings in men or women with dementia have been inconsistent. For example, one study suggested that women with Alzheimer’s have a similar prevalence of depression as men, despite a higher prevalence among women without Alzheimer’s disease (Tremblay et al., 2023). In contrast, a systematic review and meta-analysis of 62 studies found that women with Alzheimer’s disease have a higher prevalence of depression than men (Eikelboom et al., 2022). It is possible that men without dementia are less likely than women to visit clinics and receive a depression diagnosis, while men with dementia may have similar chances of diagnosis as women. Similarly, detection of depression among men in this study may be improved because all participants were required to report their depressive symptoms using validated scales with defined cutoffs.

Our subgroup analyses also showed that people with newly occurred dementia had a significantly higher subsequent risk of depression among those younger than 65 years and those who were 75 years or older, but not among those between 65 and 74 years, although the associations were significant for cognitive impairment in all three age groups. This differs from findings in the Swedish population (Crump et al., 2024), although prior studies have been inconsistent, as some suggested that younger age is more predictive of depression among dementia patients (Steck et al., 2018), and others finding no age-group differences (Crump et al., 2024). Some evidence has suggested that neuropsychiatric symptoms are more common in patients with early-onset dementia (Fisher et al., 2024), although further elucidation. specifically of depression risk after newly occurred dementia. is needed. It is possible that adults aged ≥65 with more advanced impairment may be less likely to endorse negative affect items, which could limit the detection of depression compared with adults <65 (Devita et al., 2022). Meanwhile, people who were 75 or older with dementia might suffer from additional physical conditions, which could be reflected in somatic depressive symptoms (e.g., “everything was an effort,” “sleep was restless,” “could not get going”) (Trivedi, 2004).

In addition, our results showed that newly occurred dementia and cognitive impairment are associated with a higher risk of depression in both non-Hispanic White and other races and ethnicities, which suggested the associations are generalizable, and newly occurred dementia and cognitive impairment may be widely used to predict subsequent risk of depression.

Our study has important implications for clinical medicine and public health. We found that individuals with cognitive impairment face an immediate elevated risk of depression, which emphasizes the need for early evaluation and prevention given the severe consequences of late-life depression (Wei et al., 2019). Prior research suggests that depression and dementia may interact synergistically to worsen subsequent health outcomes in both community and clinical populations (Bellelli et al., 2008; Olofsson et al., 2023; Perna et al., 2019), further highlighting the importance of timely prevention or treatment. It is notable that our findings indicate that depression risk was significantly elevated not only in people with dementia but also in those with cognitive impairment. This suggests that early detection of cognitive impairment, even before dementia develops, provides a crucial opportunity for intervention.

Clinicians should proactively screen for depressive symptoms among patients with dementia or cognitive impairment using validated instruments selected according to cognitive severity, ability to self-report, and language. For patients with mild cognitive impairment or preserved ability to self-report, brief instruments such as the Patient Health Questionnaire-2 followed by the Patient Health Questionnaire-9, or the Geriatric Depression Scale, may be appropriate (Kroenke et al., 2001, 2003; Levis et al., 2020). For patients with dementia, particularly when self-report is unreliable, informant-assisted or clinician-rated instruments such as the Cornell Scale for Depression in Dementia may be preferable (Alexopoulos et al., 1988). Screening should be implemented with systems in place for diagnostic confirmation, treatment or referral, and follow-up (Barry et al., 2023). For patients who do not speak English, clinicians should use linguistically and culturally validated translated versions of these instruments when available, administered by trained bilingual staff or professional medical interpreters rather than by ad hoc translation, with attention to language-specific validation studies and cut points (“ITC Guidelines for Translating and Adapting Tests (Second Edition),” 2018).

In addition, interdisciplinary collaboration should be operationalized through approaches of collaborative care, including warm handoffs, electronic consultation, regular case conferences, and stepped care protocols. In such models, primary care clinicians and geriatricians can conduct routine screening and longitudinal monitoring, neurologists or dementia specialists can clarify cognitive diagnosis and severity, psychiatrists or behavioral health clinicians can guide management of complex or persistent depression, and a care manager, nurse, or social worker can coordinate referrals, caregiver engagement, community resources, and follow-up (Callahan et al., 2006; Possin et al., 2019). At the health system level, infrastructure can support depression prevention and screening by embedding validated depression screening into dementia care workflows; using electronic health record prompts, registries, and quality metrics to ensure diagnostic evaluation, referral or treatment, and follow-up; supporting collaborative care and dementia care-navigation models; training clinicians and community health workers; ensuring access to interpreters and validated translated tools; and strengthening community-clinical linkages with aging services, caregiver support programs, and social services to address barriers such as isolation, transportation, and caregiver strain.

Our study has several limitations. First, dementia was assessed using cutoffs from cognitive tests, and depression was based on the short CES-D scale rather than clinical diagnoses or a dementia-specific neuropsychiatric assessment. Although the eight-item CES-D has been validated and widely used in HRS, the accuracy and reliability of self-reported depressive symptoms may be affected by cognitive impairment, particularly among participants with more advanced dementia who may have reduced insight, recall difficulty, or communication limitations. In HRS, CES-D items are self-reported and are not asked of proxy respondents. Therefore, depressive symptoms may be misclassified or under-ascertained among participants who were unable to complete self-reports. Our findings should therefore be interpreted as risk of elevated depressive symptoms measured by the CES-D rather than clinically diagnosed major depression. However, the CES-D was the best available measure for this analysis because it was collected repeatedly across HRS waves, whereas structured diagnostic interviews or dementia-specific depression instruments were not available longitudinally for all participants. This may lead to some misclassification, although both the Langa-Weir Classification for dementia and the 8-item CES-D for depression have been shown to have high validity (Glymour et al., 2012; Steffick, 2000; Turvey et al., 1999). Most covariates were also based on self-report. Second, because depression was defined using the 8-item CES-D rather than a structured psychiatric assessment or BPSD-specific instrument, we could not distinguish depressive symptoms occurring as neuropsychiatric symptoms of dementia from major depressive disorder, adjustment-related symptoms, or other overlapping clinical presentations. Future studies using detailed neuropsychiatric assessments may help clarify the extent to which post-dementia depressive symptoms represent BPSD versus co-occurring depressive disorders. In addition, confounding is possible from unmeasured variables such as diet or other behavioral factors. Despite these limitations, the HRS provides a large, nationally representative cohort with long-term biennial follow-up and a comprehensive set of covariates, allowing us to examine the long-term risk of depression among people with newly occurred dementia and cognitive impairment in the United States.

In sum, in a nationally representative cohort, newly occurred dementia and cognitive impairment were associated with immediate and sustained elevated risk of depression across 10 years of follow-up, and the associations were significant in most subgroups. The findings support timely depression screening and management following cognitive decline and motivate research on preventive interventions.

## Supplementary Material

igag074_Supplementary_Data

## Data Availability

The Health and Retirement Study data can be accessed at https://hrs.isr.umich.edu/data-products. The study was not preregistered.
