# Supplementary material for: Risk of depression after new-onset dementia or cognitive impairment: the Health and Retirement Study
Source: Innov Aging. 2026 Jun 30;10(9):igag074. doi: 10.1093/geroni/igag074 (PMC13461191; doi:10.1093/geroni/igag074)
Supplement: Supplementary file 1 [file igag074_supplementary_data.pdf]

Innovation in Aging **Supplementary Material: Wei, Kim, Tang, & Crump. Risk of depression after new-onset dementia or cognitive impairment: The Health and Retirement Study**

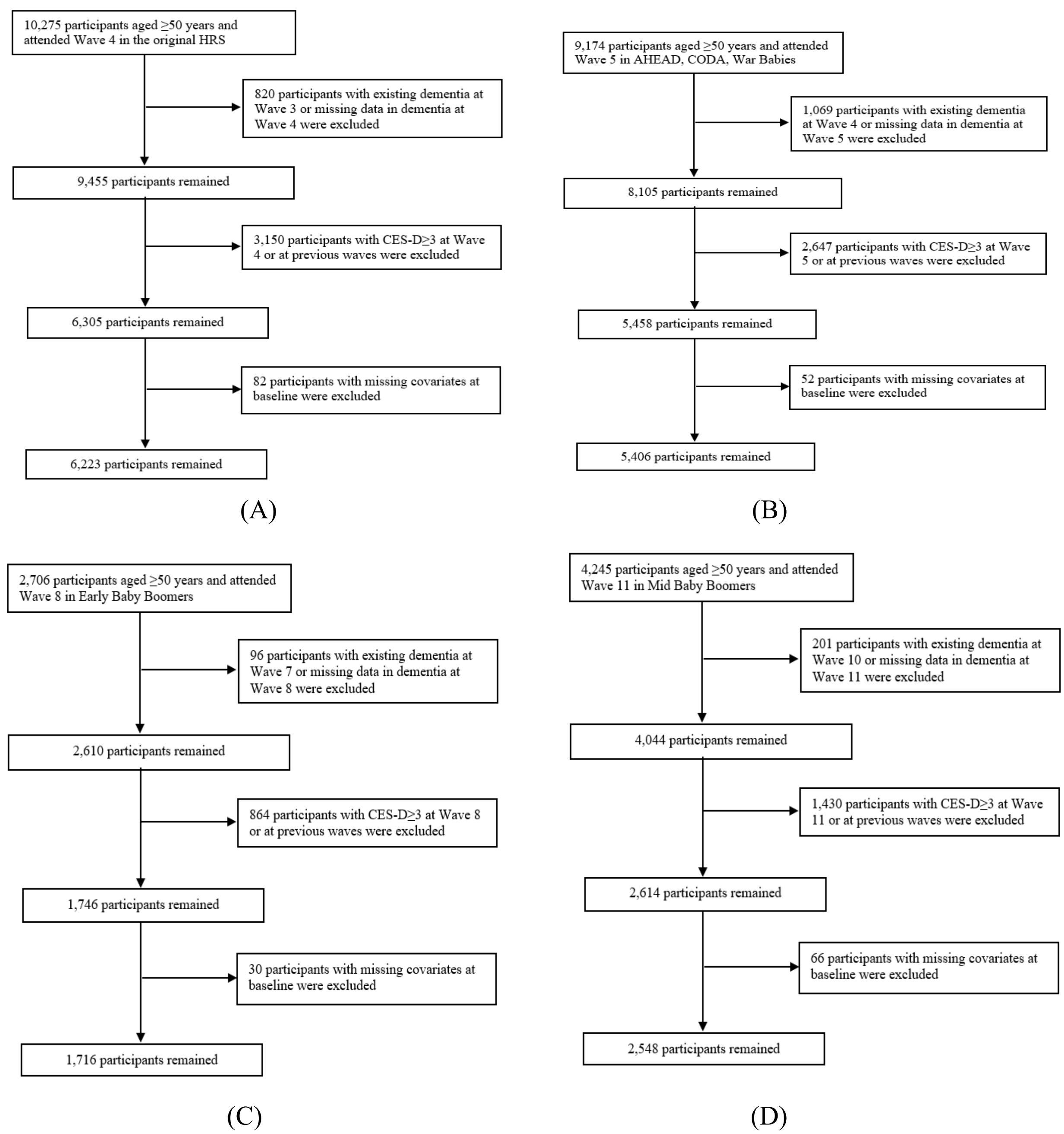

Supplementary Figure 1. Selection of participants from (A) 1998 (original HRS), (B) 2000 (AHEAD, CODA, War Babies), (C) 2006 (Early Baby Boomers), (D) 2012 (Mid Baby Boomers) for comparison of newly occurred dementia vs. no dementia.

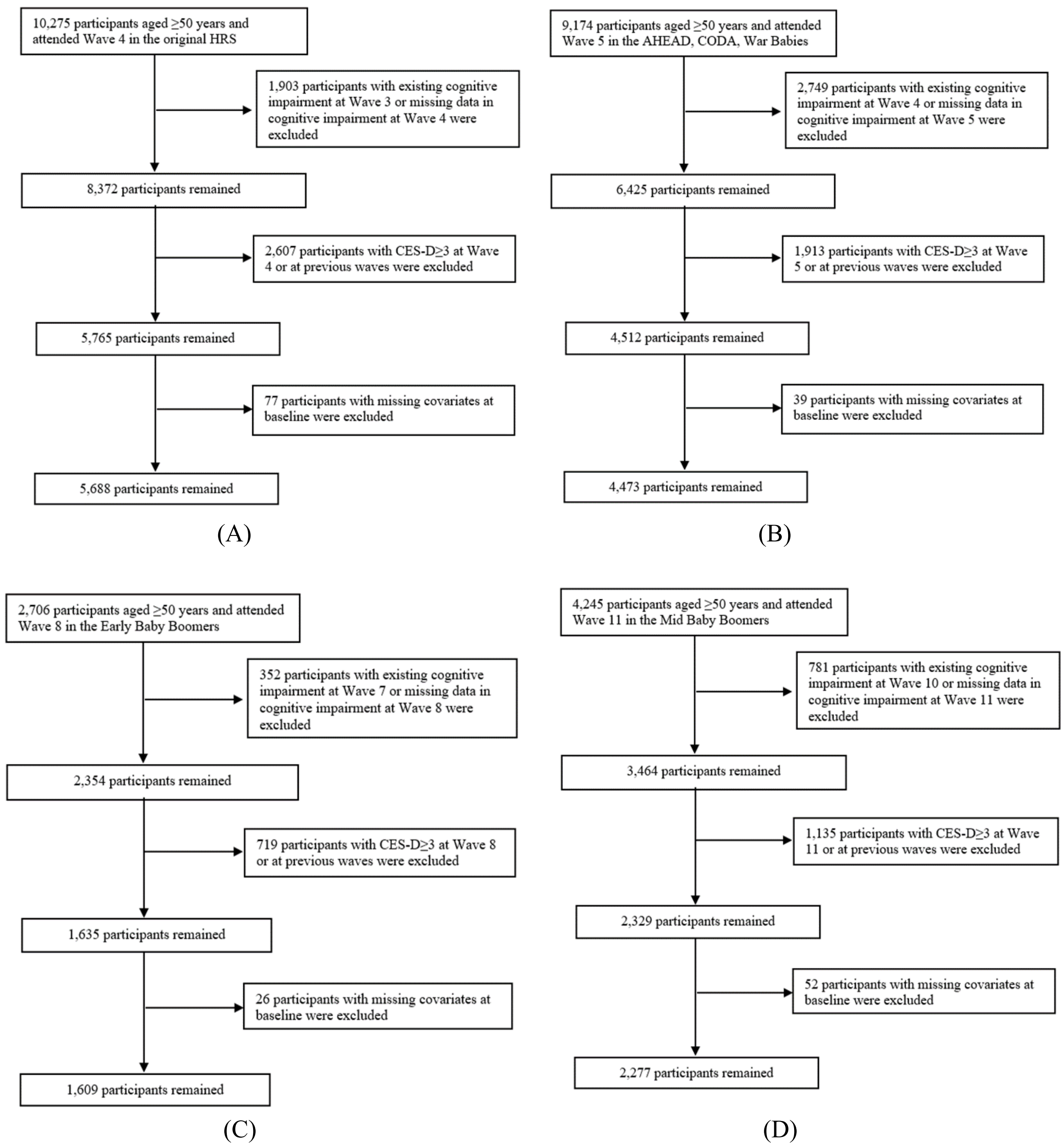

Supplementary Figure 2. Selection of participants from (A) 1998 (original HRS), (B) 2000 (AHEAD, CODA, War Babies), (C) 2006 (Early Baby Boomers), (D) 2012 (Mid Baby Boomers) for comparison of newly occurred cognitive impairment vs. no cognitive impairment

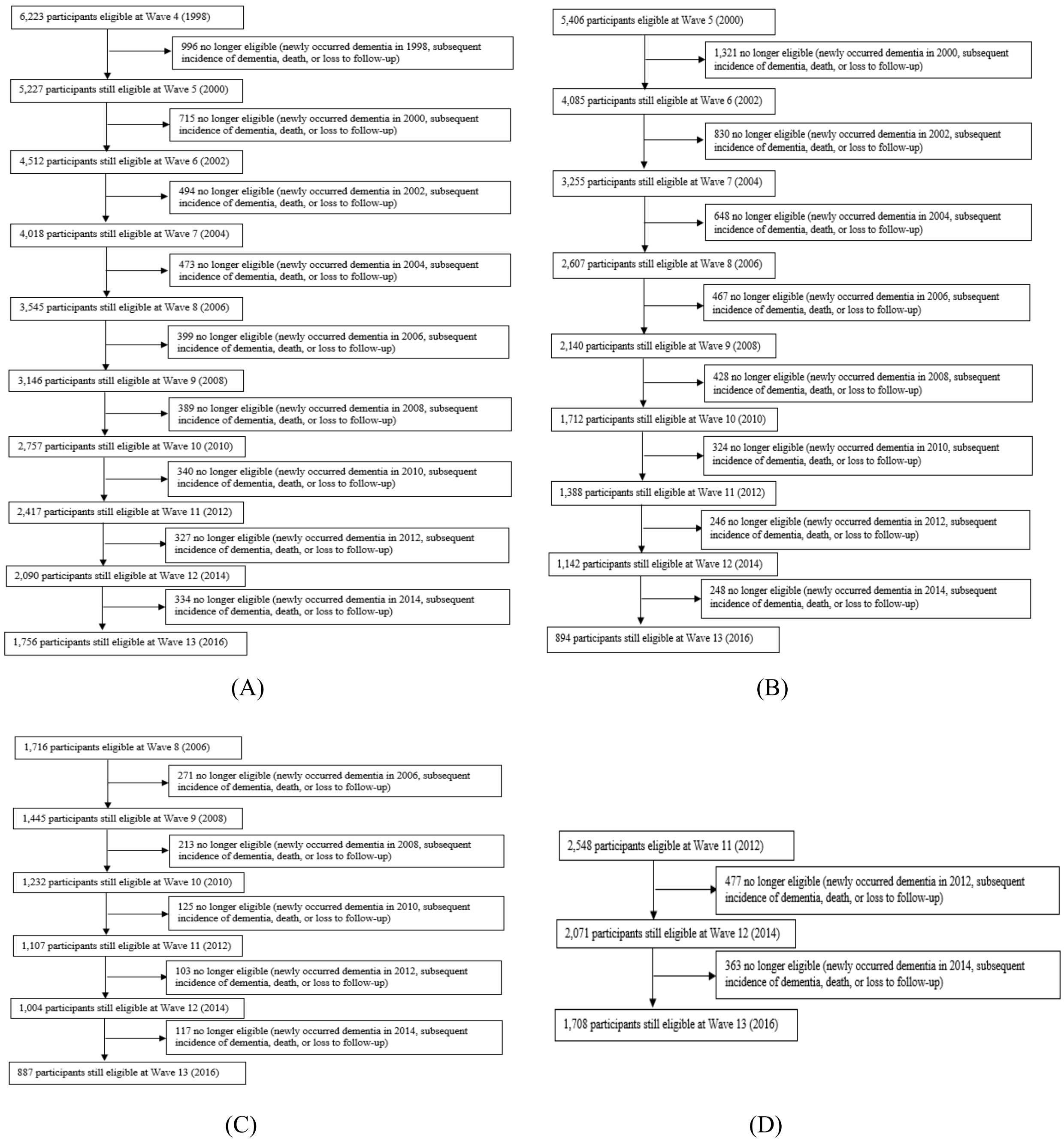

Supplementary Figure 3. Multiple eligibility for newly occurred dementia from (A) 1998 (original HRS), (B) 2000 (AHEAD, CODA, War Babies), (C) 2006 (Early Baby Boomers), (D) 2012 (Mid Baby Boomers) for comparison of newly occurred dementia vs. no dementia

Supplementary Figure 4. Multiple eligibility for newly occurred dementia from (A) 1998 (original HRS), (B) 2000 (AHEAD, CODA, War Babies), (C) 2006 (Early Baby Boomers), (D) 2012 (Mid Baby Boomers) for comparison of newly occurred cognitive impairment vs. no cognitive impairment

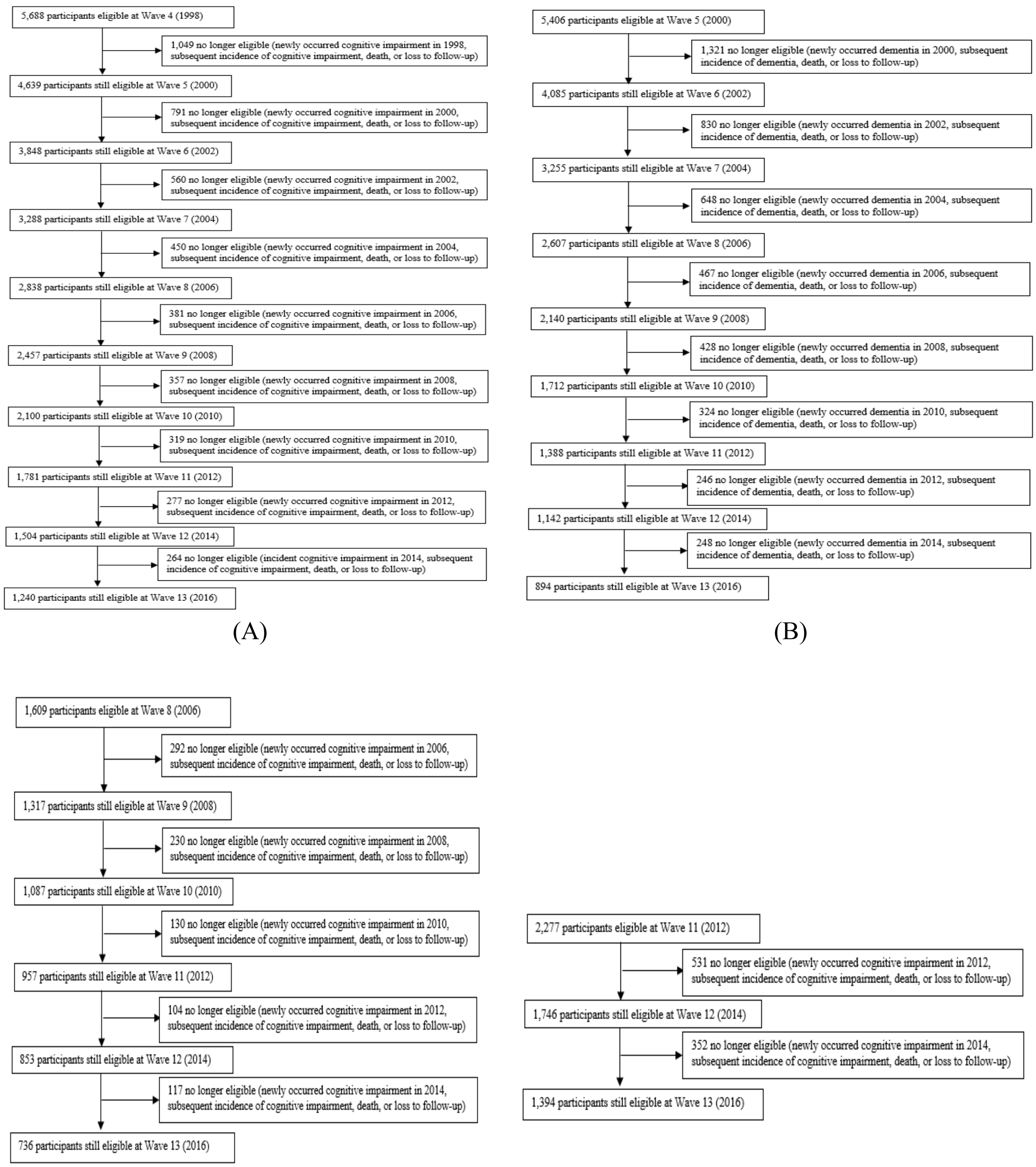

(A) (B)

(C) (D)

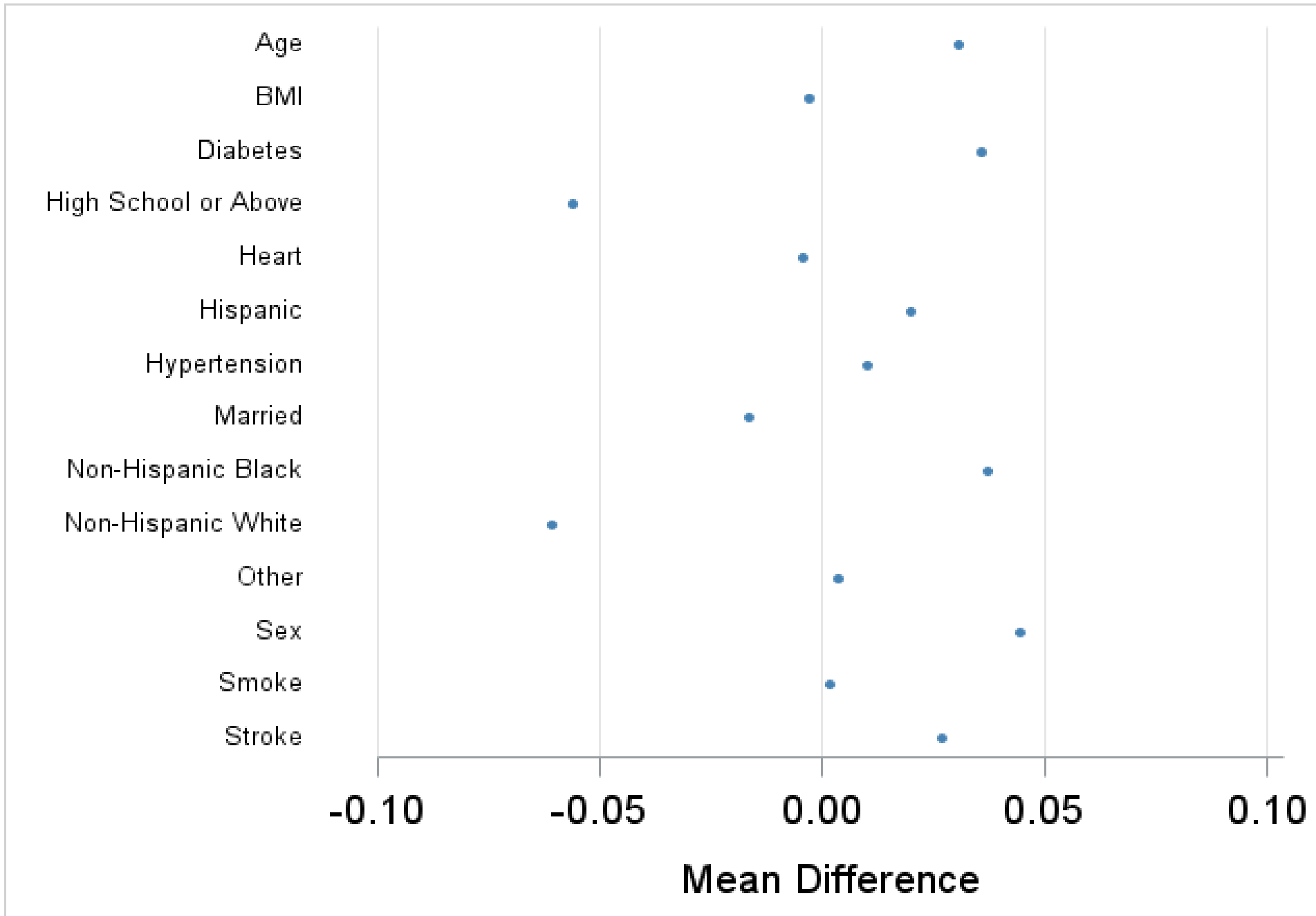

(A)

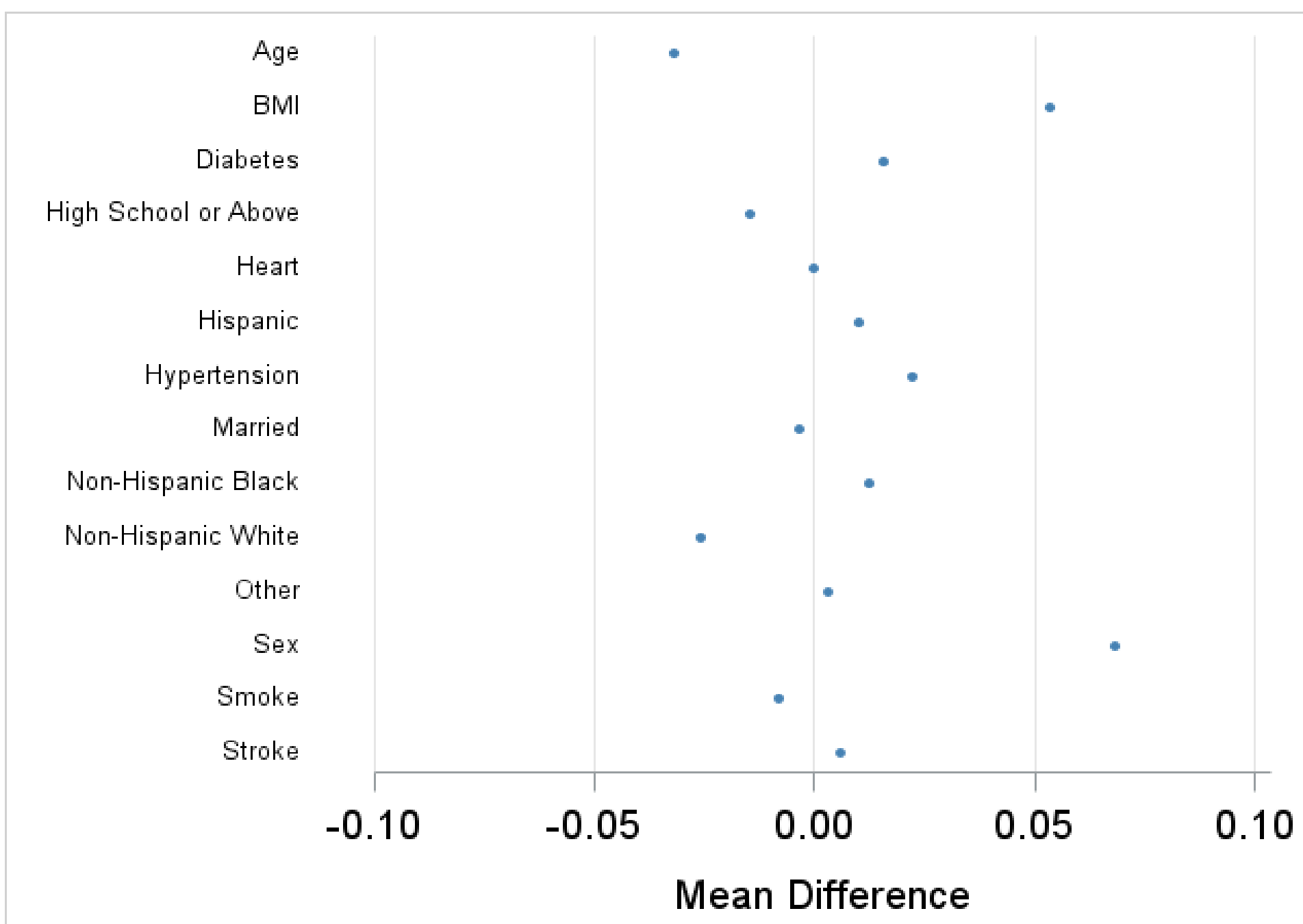

(B)

Supplementary Figure 5. Standardized mean difference of baseline characteristics for comparison of (A) newly occurred dementia vs. no dementia, and (B) newly occurred cognitive impairment vs. no cognitive impairment

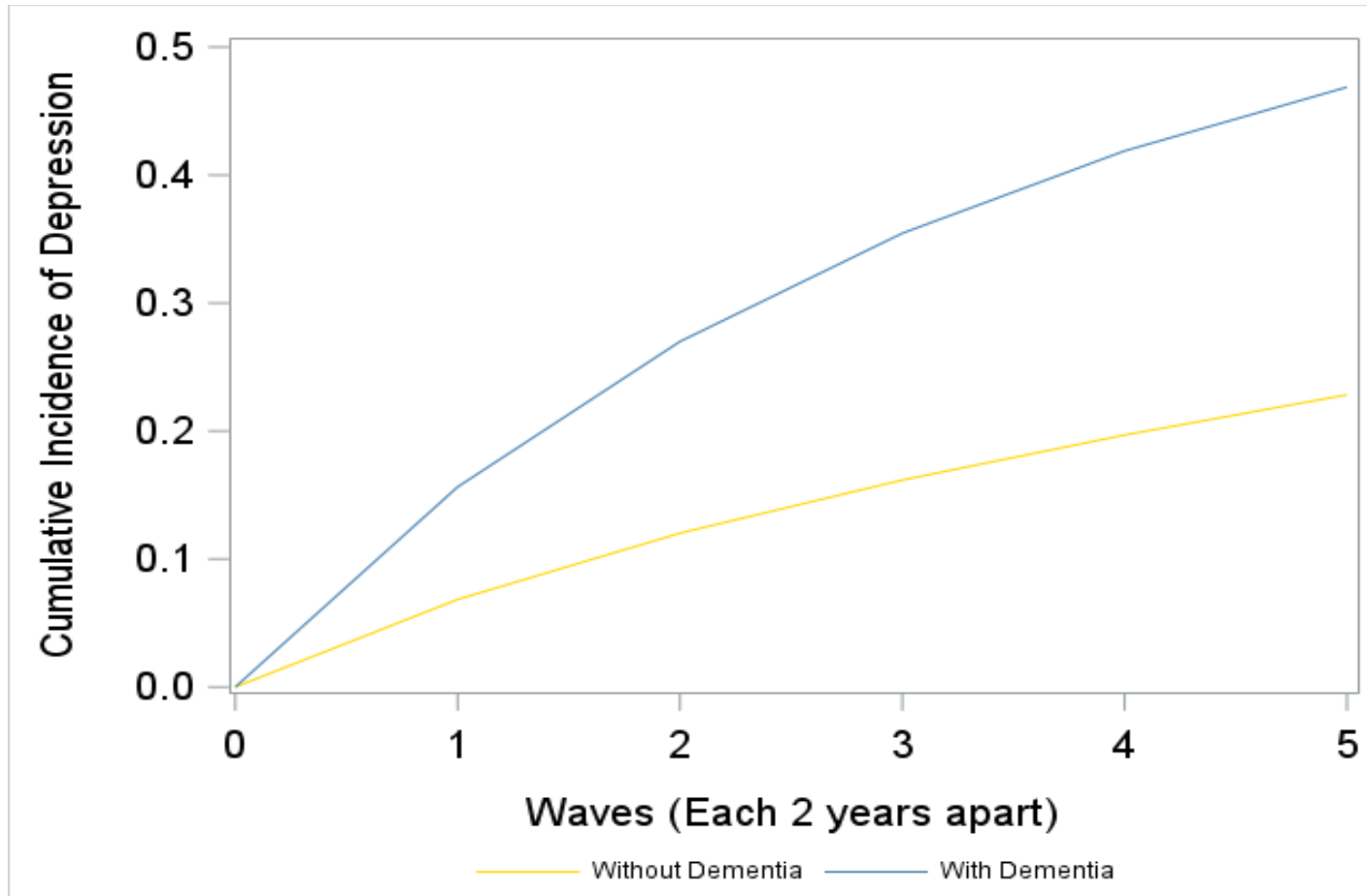

(A)

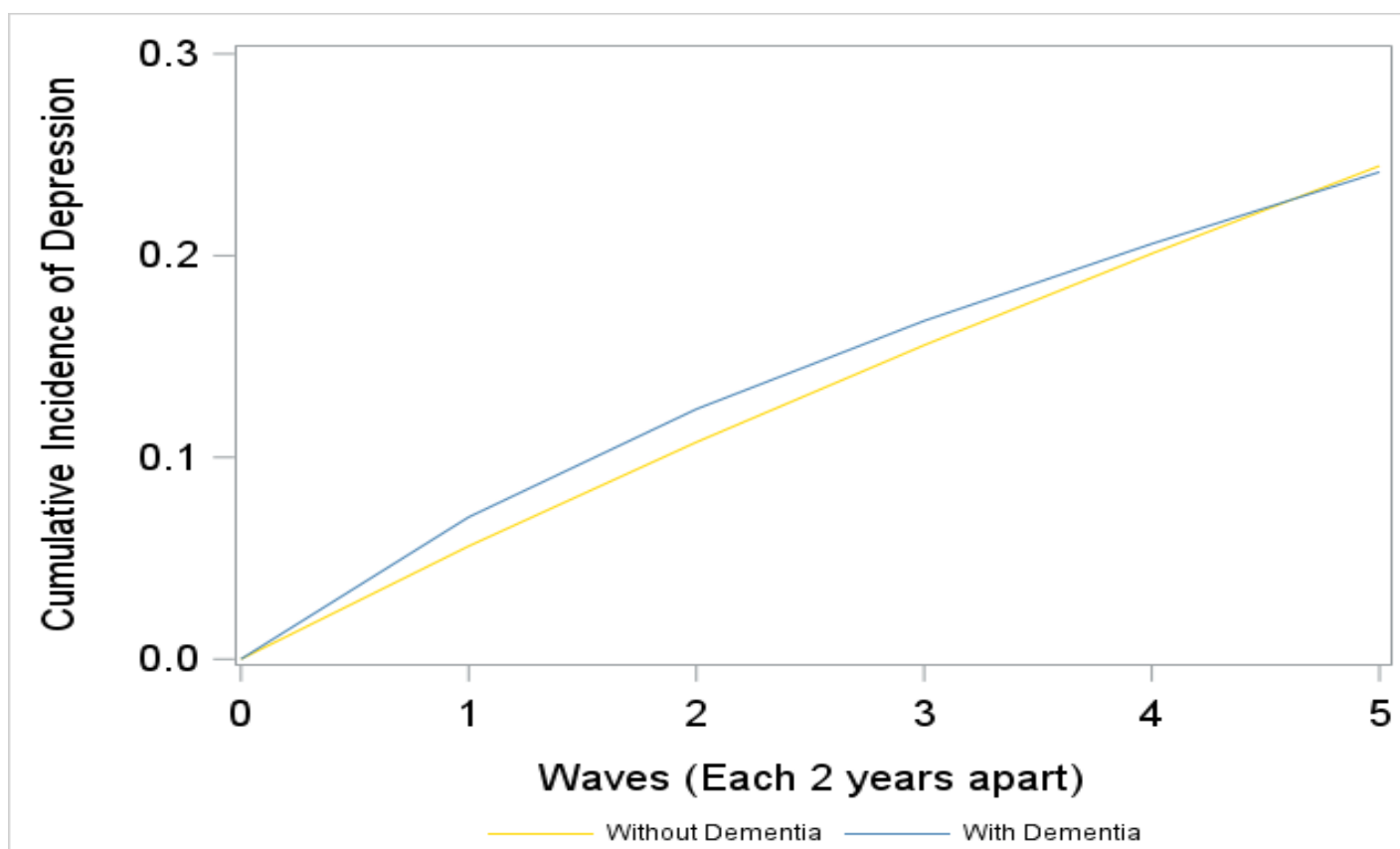

(B)

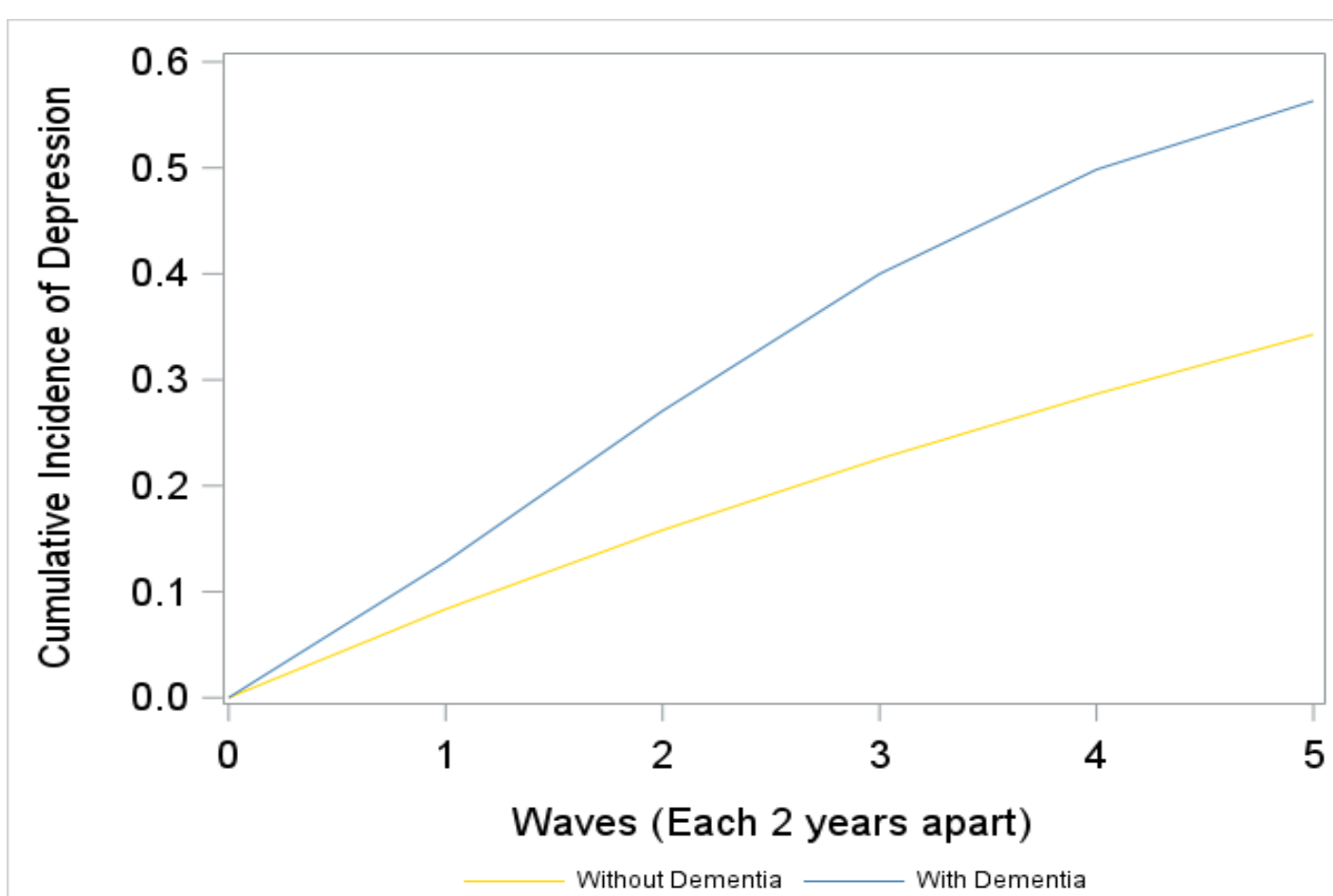

(C)

Supplementary Figure 6. Cumulative incidence of depression over 10 years of follow-up among adults aged (A) <65 years, (B) 65-74 years, or (C) ≥75 years with newly occurred dementia

Supplementary Table 1. Cumulative incidence and risk ratios for depression following newly occurred dementia among people aged <65, 65-74 or ≥75 years

**People <65**	**Length of follow-up**	**1-survival among people without dementia**	**1-survival among people with newly occurred dementia**	**Risk ratio (95% CI)**
	2 years	0.07	0.16	2.28 (0.99, 3.41)
	4 years	0.12	0.27	**2.25 (1.25, 3.07)**
	6 years	0.16	0.35	**2.19 (1.41, 2.86)**
	8 years	0.20	0.42	**2.13 (1.49, 2.71)**
	10 years	0.23	0.47	**2.05 (1.47, 2.58)**
**People 65-74**	**Length of follow-up**	**1-survival among people without dementia**	**1-survival among people with newly occurred dementia**	**Risk ratio (95% CI)**
	2 years	0.06	0.07	1.26 (0.45, 1.96)
	4 years	0.11	0.12	1.15 (0.52, 1.74)
	6 years	0.16	0.17	1.08 (0.55, 1.61)
	8 years	0.20	0.21	1.02 (0.57, 1.49)
	10 years	0.24	0.24	0.99 (0.59, 1.39)
**People ≥75**	**Length of follow-up**	**1-survival among people without dementia**	**1-survival among people with newly occurred dementia**	**Risk ratio (95% CI)**
	2 years	0.08	0.13	**1.53 (1.07, 1.89)**
	4 years	0.16	0.27	**1.71 (1.38, 2.07)**
	6 years	0.23	0.40	**1.77 (1.44, 2.13)**
	8 years	0.29	0.50	**1.74 (1.39, 2.05)**
	10 years	0.34	0.56	**1.64 (1.28, 1.94)**

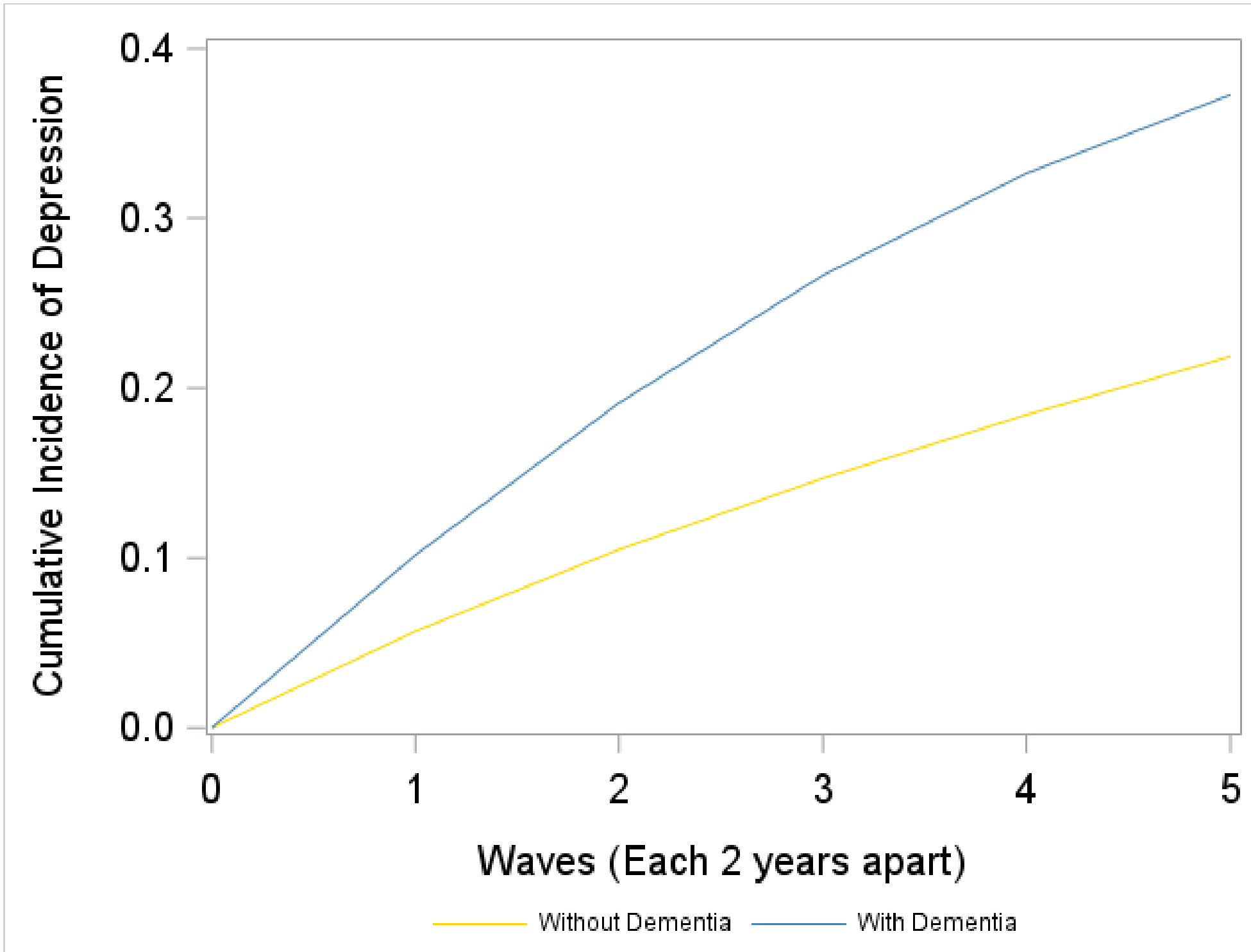

(A)

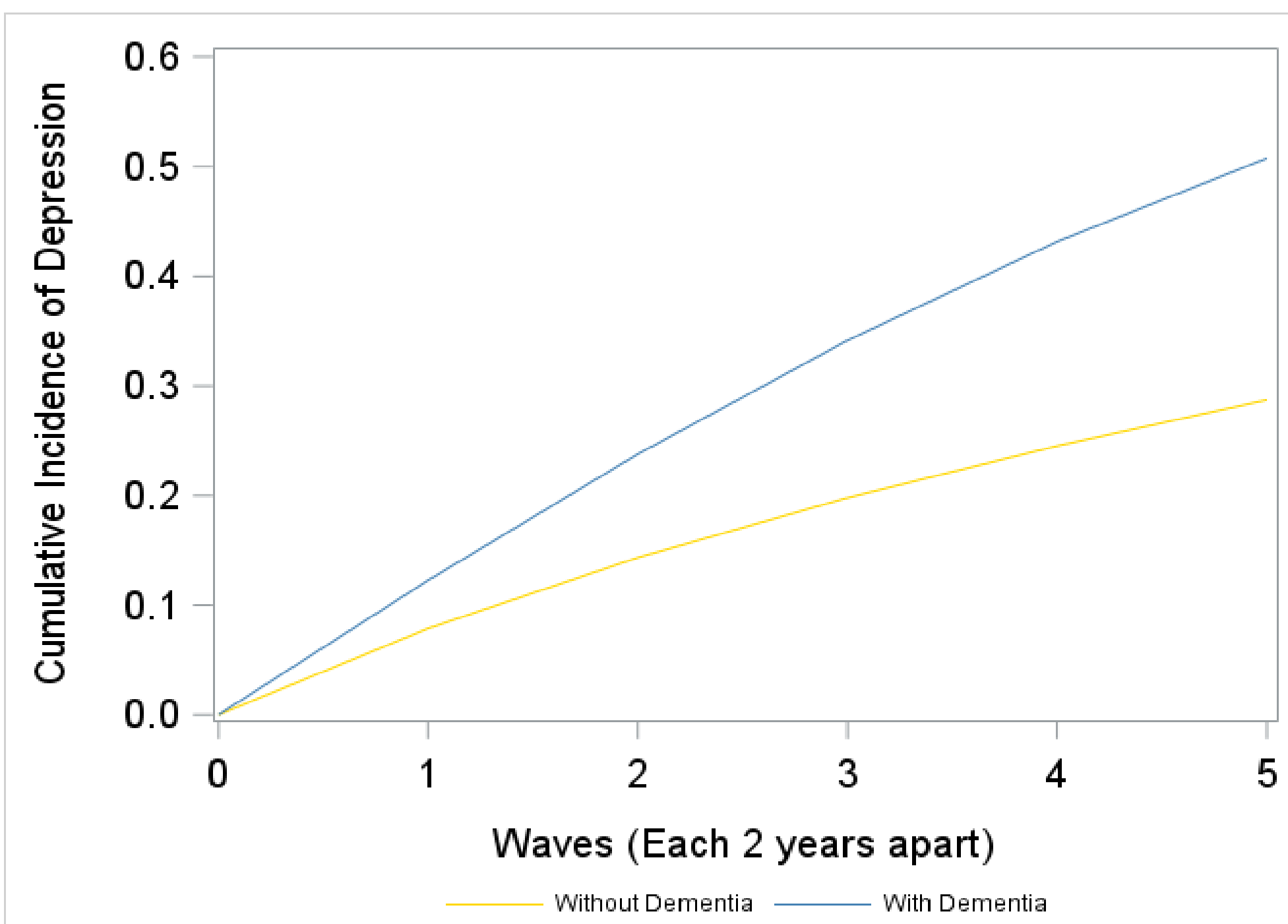

(B)

Supplementary Figure 7. Cumulative incidence of depression during 10 years of follow-up among (A) men and (B) women with newly occurred dementia

Supplementary Table 2. Cumulative incidence and risk ratios for depression following newly occurred dementia among men and women

**Men**	**Length of follow-up**	**1-survival among people without dementia**	**1-survival among people with newly occurred dementia**	**Risk ratio (95% CI)**
	2 years	0.06	0.10	**1.79 (1.08, 2.45)**
	4 years	0.11	0.19	**1.82 (1.32, 2.39)**
	6 years	0.15	0.27	**1.81 (1.32, 2.38)**
	8 years	0.18	0.33	**1.77 (1.30, 2.31)**
	10 years	0.22	0.37	**1.70 (1.25, 2.18)**
**Women**	**Length of follow-up**	**1-survival among people without dementia**	**1-survival among people with newly occurred dementia**	**Risk ratio (95% CI)**
	2 years	0.08	0.12	1.56 (0.91, 2.17)
	4 years	0.14	0.24	**1.66 (1.15, 2.10)**
	6 years	0.20	0.34	**1.73 (1.26, 2.13)**
	8 years	0.25	0.43	**1.76 (1.34, 2.14)**
	10 years	0.29	0.51	**1.77 (1.34, 2.13)**

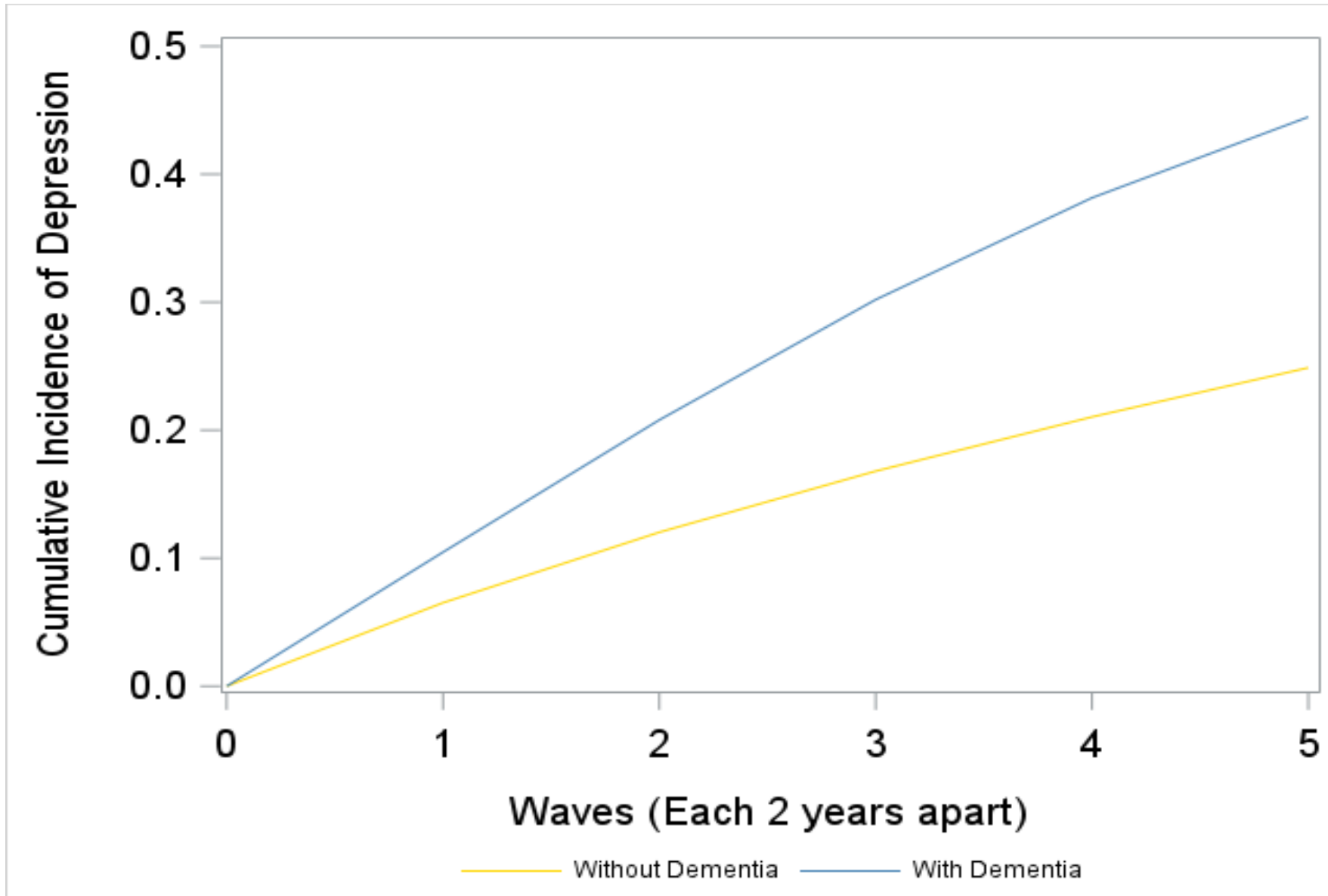

(A)

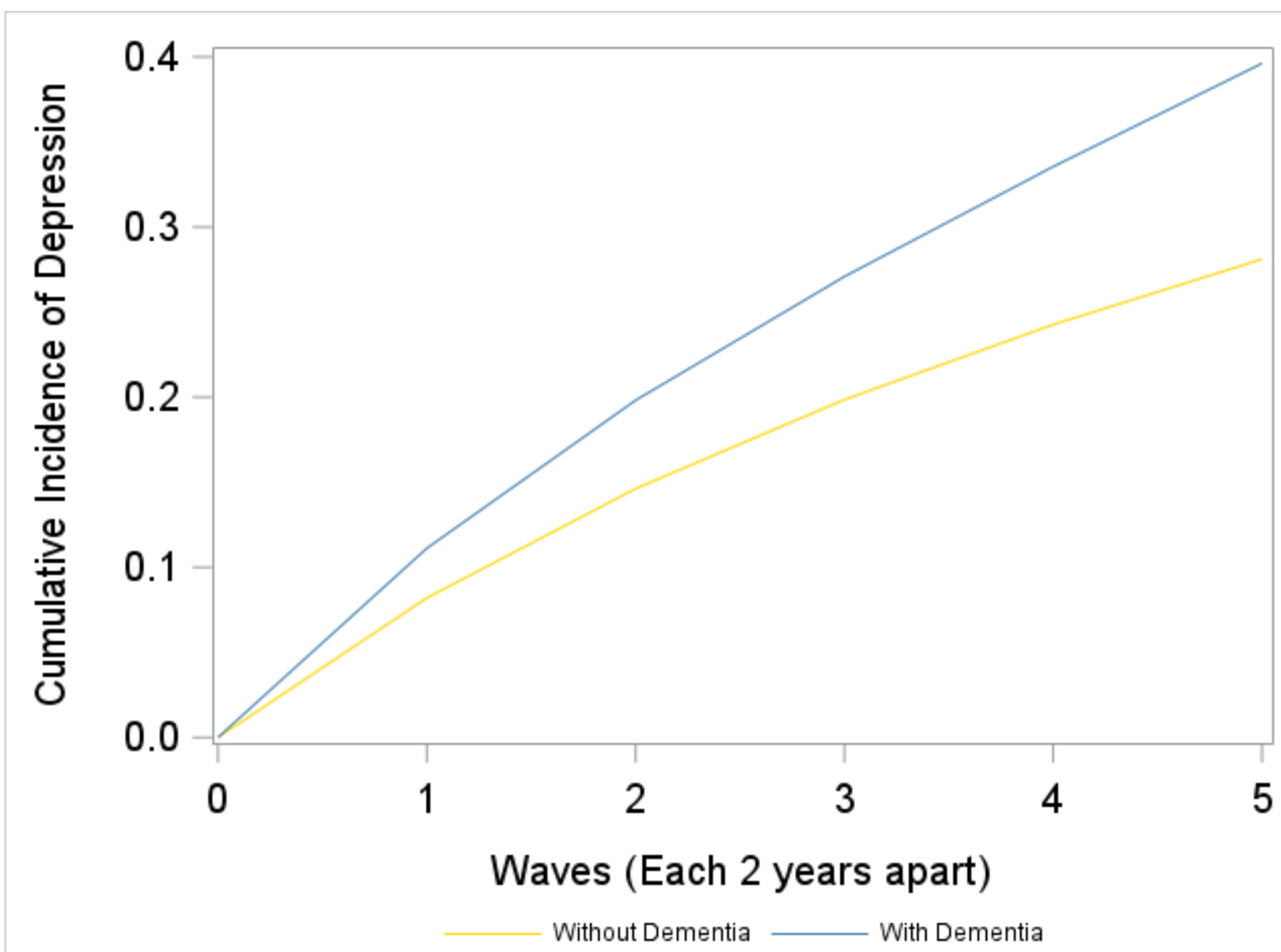

(B)

Supplementary Figure 8. Cumulative incidence of depression during 10 years of follow-up among participants of (A) non-Hispanic White and (B) other races and ethnicities with newly occurred dementia

Supplementary Table 3. Cumulative incidence and risk ratios for depression following newly occurred dementia among participants of non-Hispanic White and other races and ethnicities

**non-Hispanic White**	**Length of follow-up**	**1-survival among people without dementia**	**1-survival among people with newly occurred dementia**	**Risk ratio (95% CI)**
	2 years	0.07	0.10	1.61 (0.95, 2.24)
	4 years	0.12	0.21	**1.73 (1.19, 2.22)**
	6 years	0.17	0.30	**1.80 (1.34, 2.29)**
	8 years	0.21	0.38	**1.81 (1.37, 2.32)**
	10 years	0.25	0.44	**1.79 (1.33, 2.25)**
**Other races and ethnicities**	**Length of follow-up**	**1-survival among people without dementia**	**1-survival among people with newly occurred dementia**	**Risk ratio (95% CI)**
	2 years	0.08	0.12	1.36 (0.76, 1.86)
	4 years	0.15	0.20	1.36 (0.94, 1.75)
	6 years	0.20	0.27	**1.36 (1.02, 1.75)**
	8 years	0.24	0.34	**1.38 (1.04, 1.76)**
	10 years	0.28	0.40	**1.41 (1.05, 1.74)**

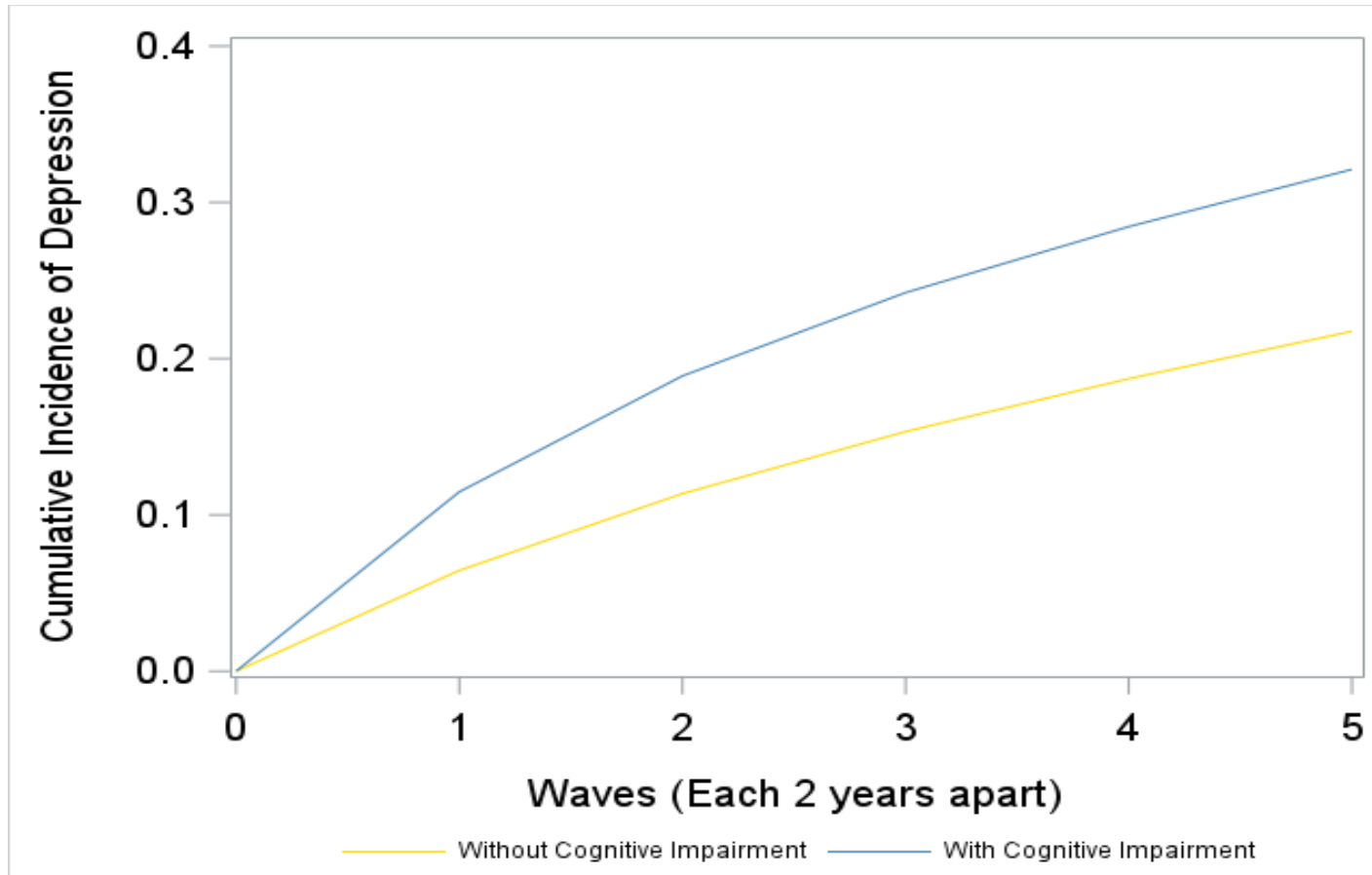

(A)

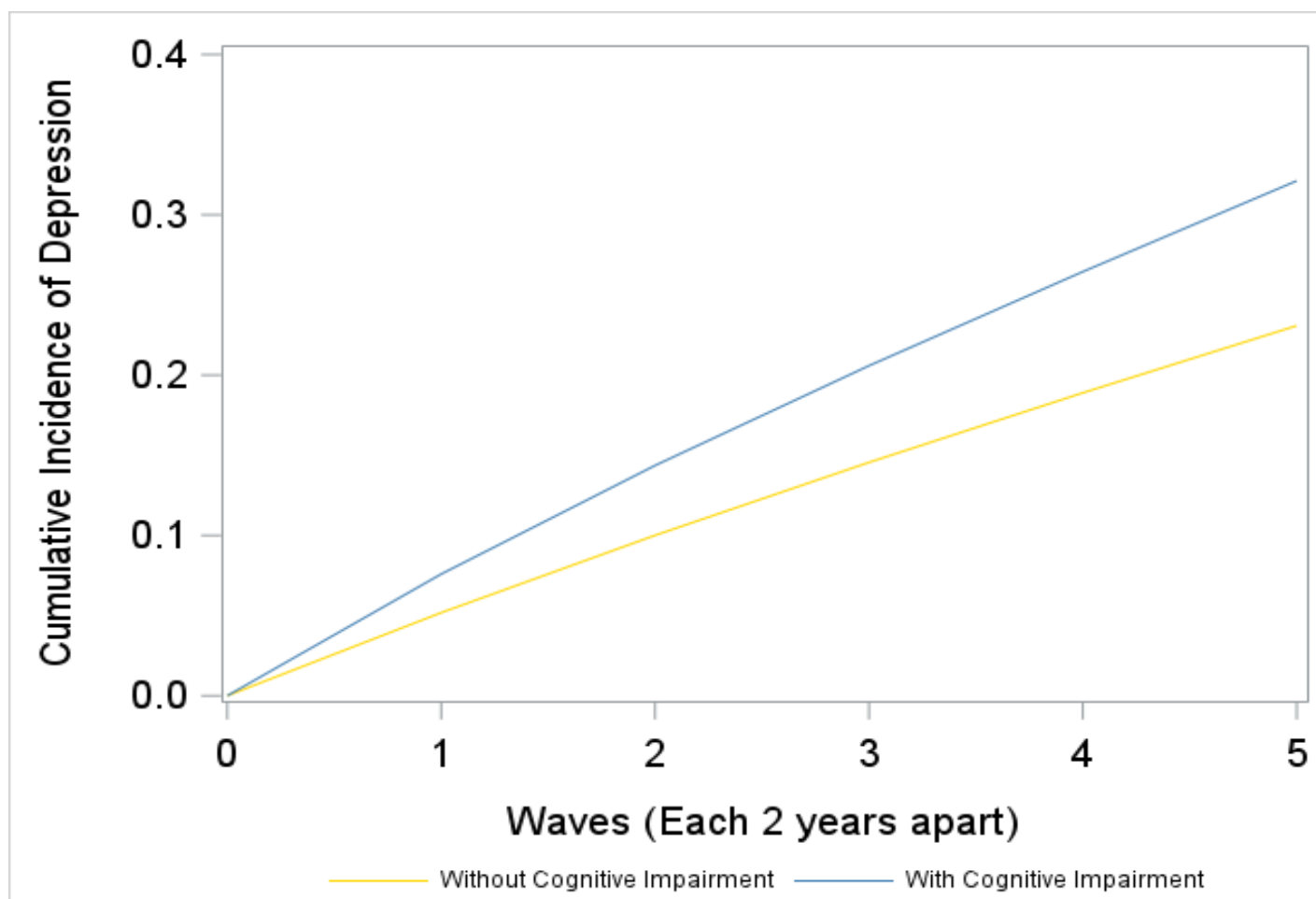

(B)

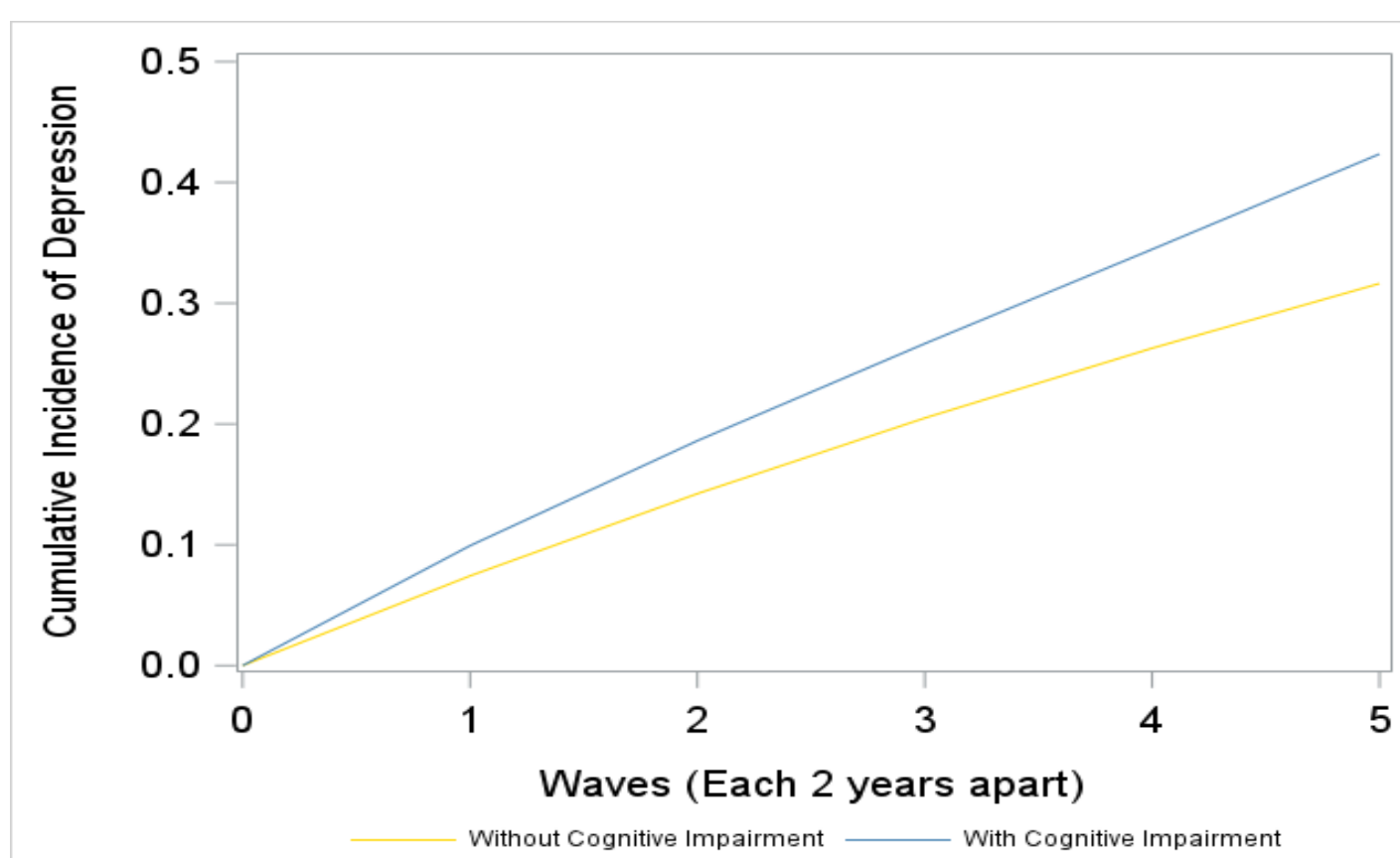

(C)

Supplementary Figure 9. Cumulative incidence of depression during 10 years of follow-up among adults aged (A) <65 years, (B) 65-74 years or (C) ≥75 years with newly occurred cognitive impairment

Supplementary Table 4. Cumulative incidence and risk ratios for depression following newly occurred cognitive impairment among people aged <65 years, 65-74 years, or ≥75 years

**People <65**	**Length of follow-up**	**1-survival among people without cognitive impairment**	**1-survival among people with newly occurred cognitive impairment**	**Risk ratio (95% CI)**
	2 years	0.06	0.11	**1.78 (1.48, 2.06)**
	4 years	0.11	0.19	**1.66 (1.44, 1.86)**
	6 years	0.15	0.24	**1.58 (1.40, 1.75)**
	8 years	0.19	0.28	**1.52 (1.36, 1.67)**
	10 years	0.22	0.32	**1.48 (1.33, 1.62)**
**People 65-74**	**Length of follow-up**	**1-survival among people without cognitive impairment**	**1-survival among people with newly occurred cognitive impairment**	**Risk ratio (95% CI)**
	2 years	0.05	0.08	**1.46 (1.17, 1.74)**
	4 years	0.10	0.14	**1.44 (1.21, 1.64)**
	6 years	0.15	0.21	**1.41 (1.24, 1.58)**
	8 years	0.19	0.26	**1.40 (1.25, 1.55)**
	10 years	0.23	0.32	**1.39 (1.26, 1.52)**
**People ≥75**	**Length of follow-up**	**1-survival among people without cognitive impairment**	**1-survival among people with newly occurred cognitive impairment**	**Risk ratio (95% CI)**
	2 years	0.07	0.10	**1.34 (1.15, 1.53)**
	4 years	0.14	0.19	**1.31 (1.18, 1.44)**
	6 years	0.20	0.27	**1.30 (1.19, 1.43)**
	8 years	0.26	0.34	**1.31 (1.20, 1.43)**
	10 years	0.32	0.42	**1.34 (1.23, 1.46)**

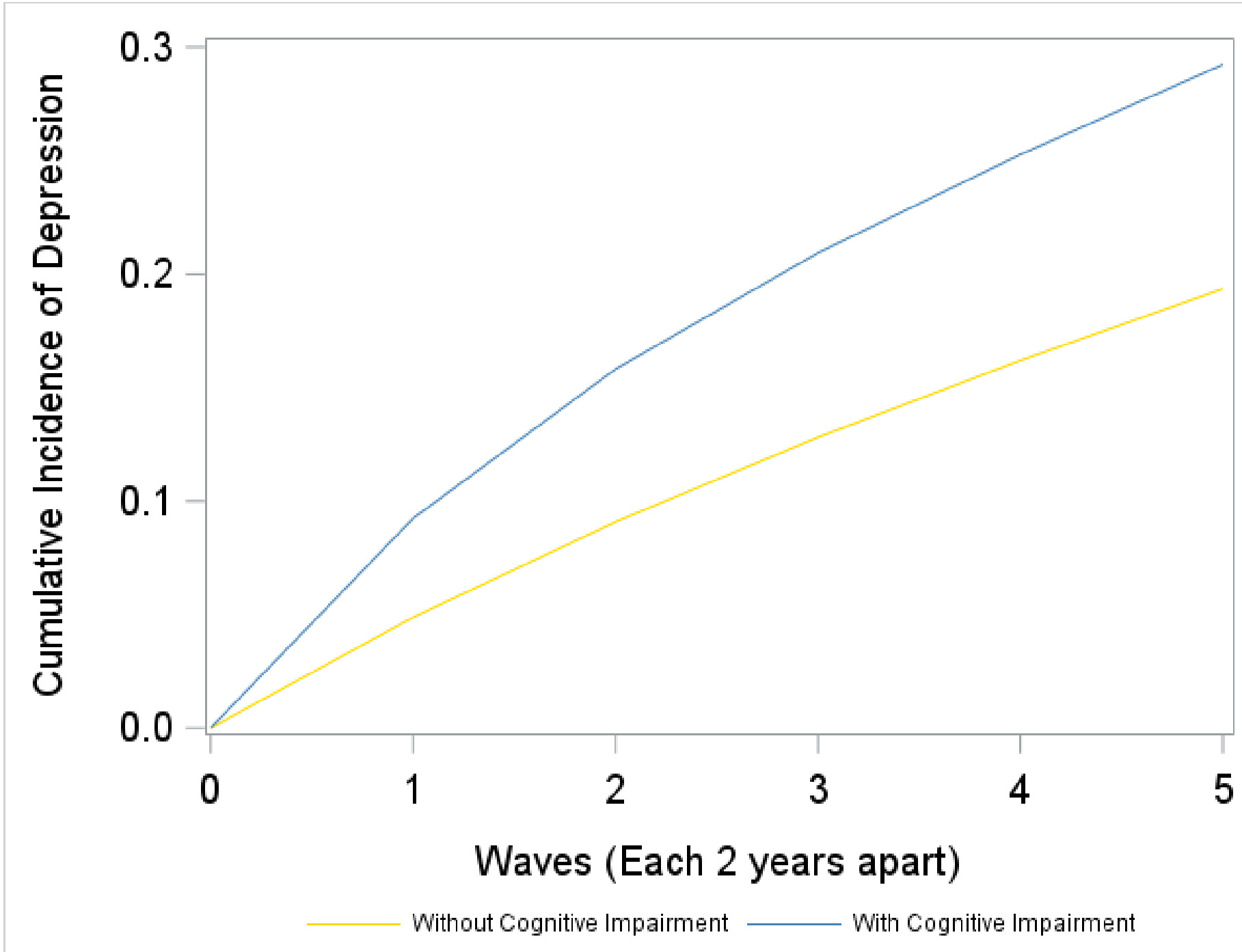

(A)

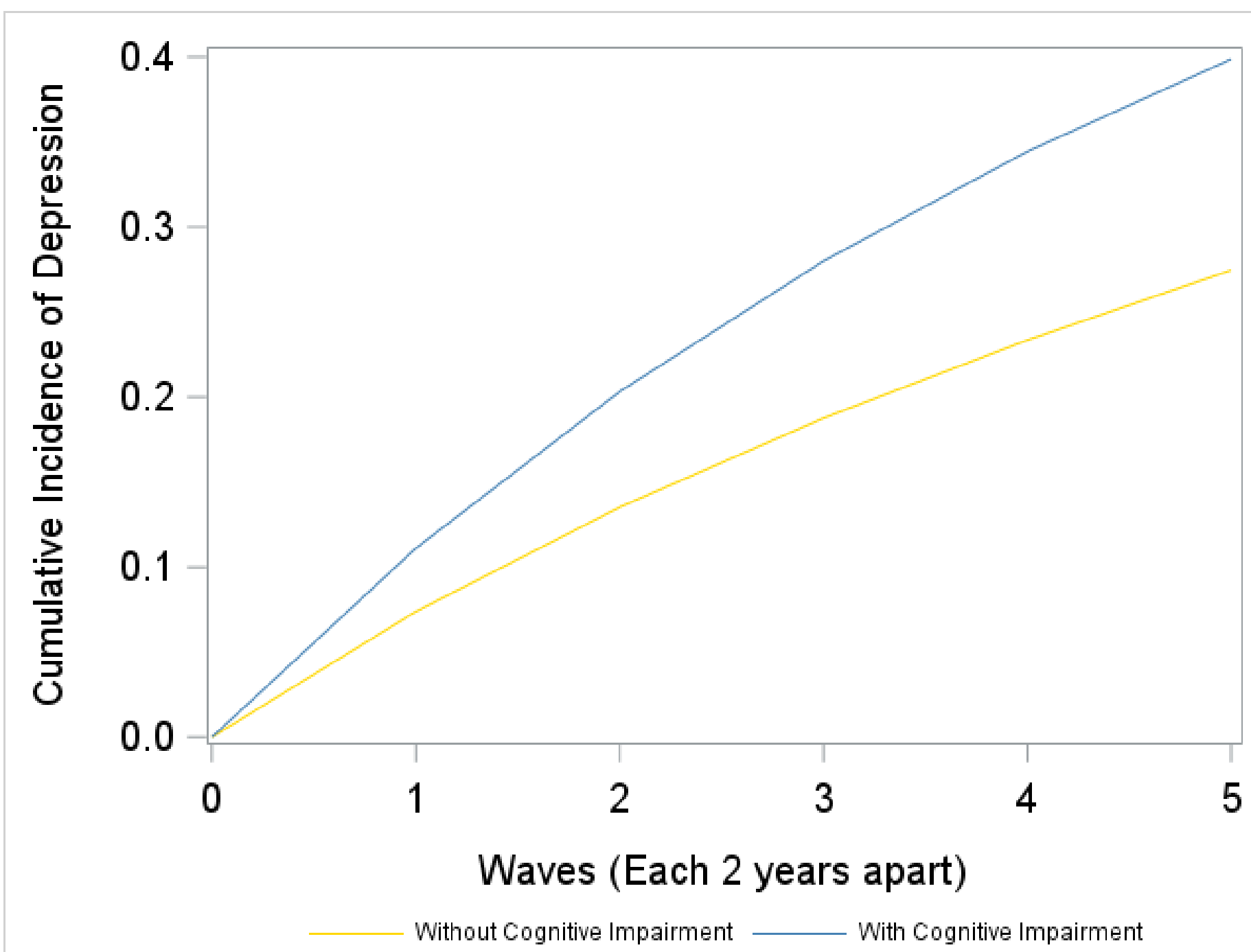

(B)

Supplementary Figure 10. Cumulative incidence of depression during 10 years of follow-up among (A) men and (B) women with newly occurred cognitive impairment

Supplementary Table 5. Cumulative incidence and risk ratios for depression following newly occurred cognitive impairment among men and women

**Men**	**Length of follow-up**	**1-survival among people without cognitive impairment**	**1-survival among people with newly occurred cognitive impairment**	**Risk ratio (95% CI)**
	2 years	0.05	0.09	**1.89 (1.64, 2.16)**
	4 years	0.09	0.16	**1.74 (1.55, 1.93)**
	6 years	0.13	0.21	**1.63 (1.48, 1.79)**
	8 years	0.16	0.25	**1.56 (1.43, 1.70)**
	10 years	0.19	0.29	**1.51 (1.39, 1.64)**
**Women**	**Length of follow-up**	**1-survival among people without cognitive impairment**	**1-survival among people with newly occurred cognitive impairment**	**Risk ratio (95% CI)**
	2 years	0.07	0.11	**1.50 (1.28, 1.74)**
	4 years	0.14	0.20	**1.50 (1.34, 1.67)**
	6 years	0.19	0.28	**1.49 (1.36, 1.63)**
	8 years	0.23	0.34	**1.48 (1.35, 1.60)**
	10 years	0.27	0.40	**1.45 (1.33, 1.57)**

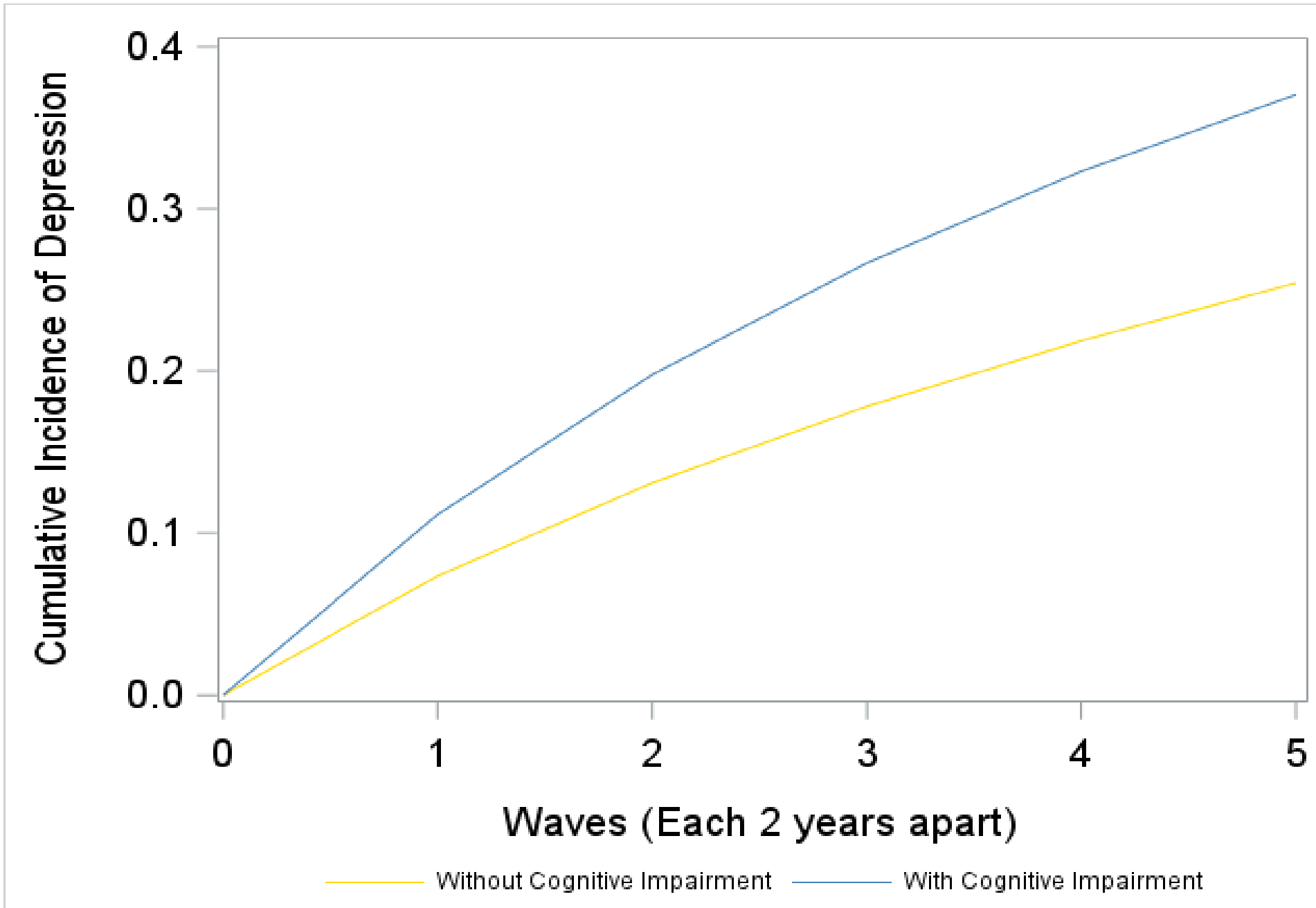

(A)

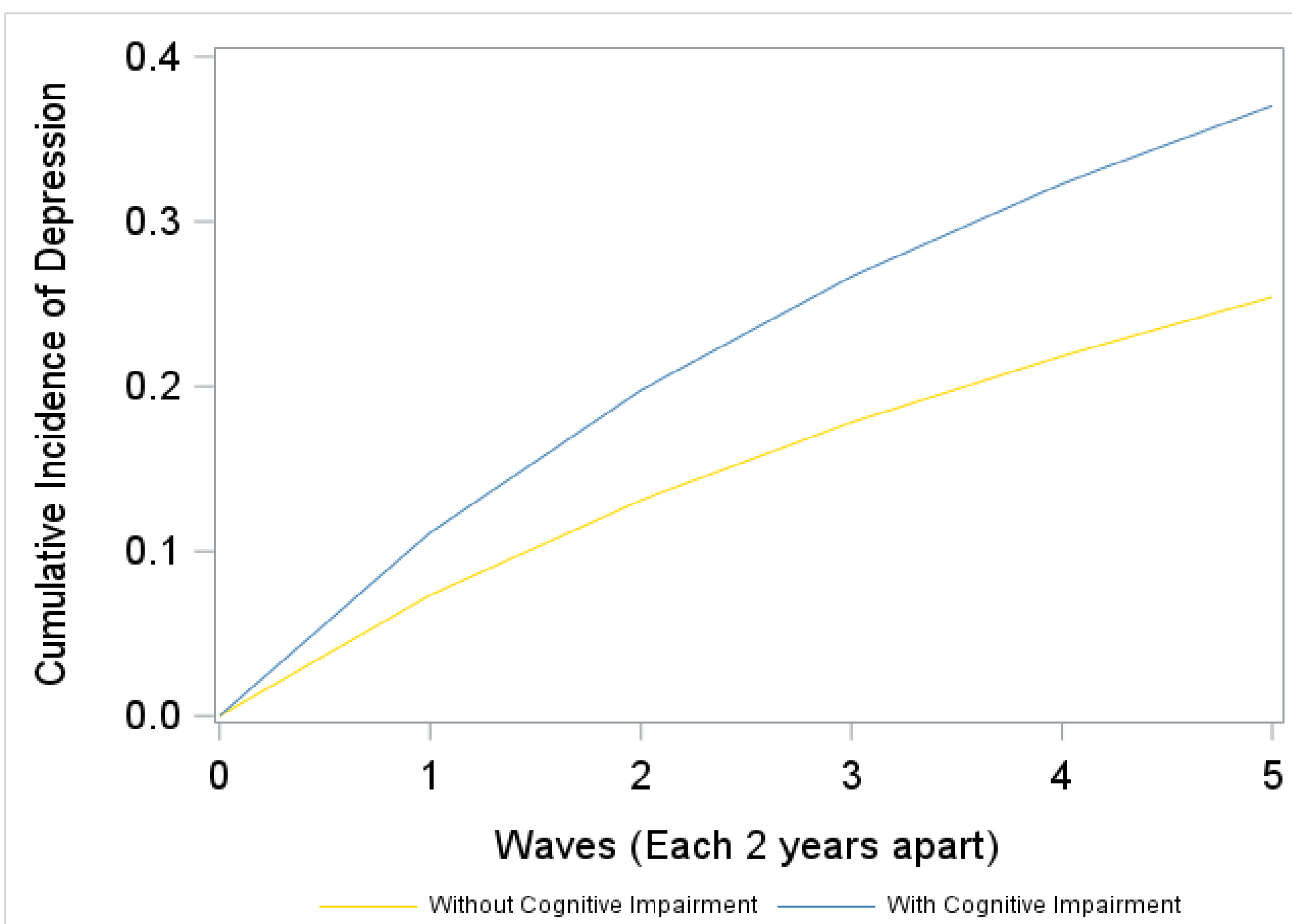

(B)

Supplementary Figure 11. Cumulative incidence of depression during 10 years of follow-up among participants of (A) non-Hispanic White and (B) other races and ethnicities with newly occurred cognitive impairment

Supplementary Table 6. Cumulative incidence and risk ratios for depression following newly occurred cognitive impairment among participants of non-Hispanic White and other races and ethnicities

**non-Hispanic White**	**Length of follow-up**	**1-survival among people without newly occurred cognitive impairment**	**1-survival among people with newly occurred cognitive impairment**	**Risk ratio (95% CI)**
	2 years	0.06	0.10	**1.63 (1.42, 1.84)**
	4 years	0.11	0.17	**1.54 (1.39, 1.71)**
	6 years	0.16	0.23	**1.48 (1.35, 1.62)**
	8 years	0.20	0.28	**1.43 (1.32, 1.56)**
	10 years	0.23	0.33	**1.40 (1.29, 1.52)**
**Other races and ethnicities**	**Length of follow-up**	**1-survival among people without newly occurred cognitive impairment**	**1-survival among people with newly occurred cognitive impairment**	**Risk ratio (95% CI)**
	2 years	0.07	0.11	**1.52 (1.27, 1.76)**
	4 years	0.13	0.20	**1.51 (1.31, 1.70)**
	6 years	0.18	0.27	**1.50 (1.33, 1.67)**
	8 years	0.22	0.32	**1.48 (1.32, 1.65)**
	10 years	0.25	0.37	**1.46 (1.31, 1.62)**
